# Effect of Endodontic Irrigants on the Cyclic Fatigue Resistance of Nickel–Titanium Rotary Instruments: A Systematic Review

**DOI:** 10.3390/ma18174056

**Published:** 2025-08-29

**Authors:** Bartłomiej Karaś, Agnieszka Kotela, Marzena Laszczyńska, Zuzanna Majchrzak, Mateusz Trafalski, Jacek Matys, Maciej Dobrzyński

**Affiliations:** 1 Medical Center of Innovation, Wroclaw Medical University, Krakowska 26, 50-425 Wroclaw, Poland; kotela.agnieszka@gmail.com (A.K.); marzenalaszczynska@gmail.com (M.L.); zuzanna.h.nawrocka@gmail.com (Z.M.); 2 Dental Surgery Department, Wroclaw Medical University, Krakowska 26, 50-425 Wroclaw, Poland; mateusz.trafalski@umw.edu.pl; 3 Department of Pediatric Dentistry and Preclinical Dentistry, Wroclaw Medical University, Krakowska 26, 50-425 Wroclaw, Poland; maciej.dobrzynski@umw.edu.pl

**Keywords:** cyclic fatigue resistance, irrigants, nickiel-titanium files, rotary endodontic instruments, sodium hypochlorite

## Abstract

Instrument fracture during endodontic treatment significantly compromises treatment outcomes, with sodium hypochlorite (NaOCl) and other irrigants potentially affecting the cyclic fatigue resistance of nickel–titanium (NiTi) rotary files. This systematic review evaluated the impact of endodontic irrigants on NiTi instrument durability. A comprehensive literature search was conducted across PubMed, Scopus, Web of Science, Embase, Cochrane Library, and WorldCat databases through June 2025, following PRISMA guidelines. Studies investigating cyclic fatigue resistance of NiTi rotary instruments exposed to various irrigants were included. Twenty-seven in vitro studies met the inclusion criteria, involving instruments across multiple file systems and irrigant solutions. The review revealed that NaOCl, particularly at concentrations ≥5% and elevated temperatures, significantly reduced cyclic fatigue resistance in most studies, with scanning electron microscopy confirming surface corrosion and microcrack formation. Heat-treated NiTi alloys demonstrated superior fatigue resistance compared to conventional austenitic alloys. Short-term NaOCl exposure (1–5 min) showed minimal impact, while prolonged exposure combined with autoclave sterilization produced cumulative weakening effects. Alternative irrigants such as EDTA and chlorhexidine showed more neutral effects on instrument integrity. These findings suggest that irrigant selection and exposure protocols significantly influence NiTi instrument longevity, with implications for clinical endodontic practice and instrument safety protocols.

## 1. Introduction

Modern dentistry is advancing across multiple disciplines at an unprecedented pace. Preventive care, restorative dentistry, and endodontics play a crucial role in preserving natural dentition and safeguarding oral health [1,2,3,4,5,6]. Long-term clinical observations, combined with the development of advanced medical imaging, have significantly deepened our understanding of the crucial role that high-quality endodontic therapy plays in achieving long-term treatment success. Continuous innovations in dental technology have markedly improved the prognosis of endodontic procedures. One of the most critical stages of root canal treatment is the precise mechanical preparation of the canal walls. Adequate canal shaping—which includes the removal of infected tissue and the creation of an optimal canal geometry—facilitates effective obturation, a key determinant of therapeutic outcome [1]. Incomplete debridement and the persistence of necrotic tissue or microbial biofilms within the root canal system can lead to complications such as residual pulpitis, periapical periodontitis, and ultimately, tooth loss [2]. Currently, the gold standard in endodontic therapy involves the mechanical preparation of root canals using rotary nickel–titanium (NiTi) instruments [3]. Advances in material science, instrument design, and clinical protocols have contributed to the widespread availability and improved performance of rotary endodontic systems.

The diversity of root canal preparation techniques has led to the development of numerous types of instruments used during endodontic treatment. Rotary instruments are classified based on their working technique: instruments designed for the step-back technique (e.g., Lightspeed), those used for the crown-down technique (e.g., Profile, Protaper), and instruments intended for the single-length technique (e.g., Mtwo) [4]. Among these, systems based on the crown-down technique are the most employed in clinical practice. A shared characteristic of all rotary systems is their construction from nickel–titanium alloy (NiTi). This alloy exhibits properties such as shape memory, flexibility, and increased fracture resistance, all of which contribute to enhanced operator comfort and improved endodontic outcomes. A key feature of rotary systems is the taper of the files. The taper may be constant along the entire file (the same difference between each millimeter of the cutting part), may vary between successive files, or may change within different sections of the same file [4,7]. Currently, most manufacturers employ computer-guided laser technology in the production process, ensuring greater precision and dimensional stability of the instruments. Depending on the intended technique, manufacturers modify parameters such as instrument shape, helical angle, cutting edge configuration, and metal alloy composition. Within a single rotary system, instruments may vary in taper, cross-section, and tip design, allowing for tailored canal shaping based on individual clinical needs. The shape of the core of the file can also influence its fatigue behavior. A larger core diameter improves resistance to torsional fatigue; however, it decreases flexibility and may shorten cyclic fatigue resistance in curved canals. Conversely, advances in heat-treatment technology have demonstrated the ability to enhance cyclic fatigue resistance, thereby extending the functional lifespan of NiTi instruments [7,8,9,10].

**Figure 1:**
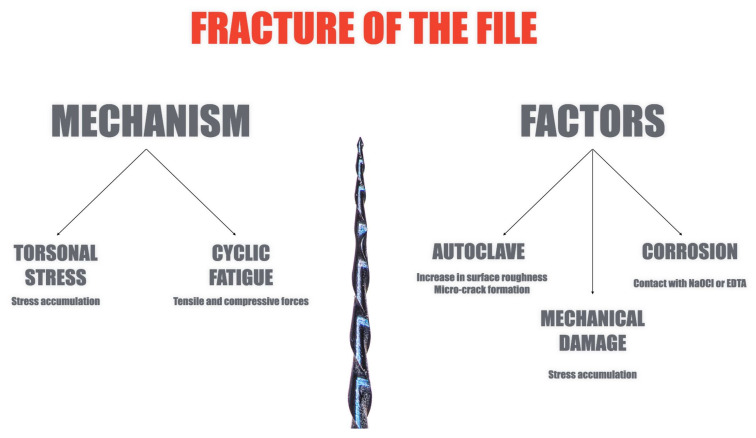
Mechanism and factors promoting fracture of the endodontic files.

Fracture of an endodontic instrument during root canal treatment constitutes a significant procedural complication that can markedly reduce the effectiveness of chemomechanical debridement, canal shaping and three-dimensional obturation of the root canal system [7,8]. Such disruption may adversely affect both the long-term prognosis and the biological outcome of endodontic therapy [9]. Instrument damage is usually attributed to two dominant mechanical failure modes—cyclic fatigue fractures and torsional stress, or combination of both [7]. Cyclic fatigue is generated by repeated tensile and compressive forces acting on the instrument as it operates in curved canals, the fracture risk increasing in anatomies featuring curvatures greater than about 30° and small radii of curvature [10,11]. Torsional failure occurs when the tip of the instrument becomes locked within the canal while the file shaft continues to rotate, leading to stress accumulation and sudden breakage [12]. Autoclave sterilization, especially after multiple cycles, can increase surface roughness and cause micro-cracks in NiTi instruments, making them more prone to damage. Routine mechanical use also creates microscopic surface defects and reduces torsional resistance, and these effects worsen when followed by sterilization. In addition, irrigants contribute to corrosion: sodium hypochlorite can cause pitting and nickel loss in NiTi files, while EDTA increases surface roughness even after short contact with the instrument [13]. These changes weaken the file structure and raise the risk of fracture. Summary of the factors is shown on Figure 1.

Existing reports usually analyze a single file system in only one or two irrigants, which makes it difficult to translate the findings into everyday practice. To date, no comparable comprehensive review or experimental comparison has been published that encompasses stainless-steel files, conventional austenitic NiTi, and modern heat-treated NiTi instruments tested against the full spectrum of clinically used irrigants—NaOCl at various concentrations, EDTA, chlorhexidine, saline, deionized water, etidronate, and lubricating oil—under fatigue test conditions, highlighting both the novelty and clinical relevance of our work. The objective of this review is to examine how the type, concentration, and exposure time of irrigants influence both the number of cycles to fracture and the post-fracture surface morphology of rotary files. From a clinical perspective, this question is critical, because file fracture can obstruct apical access, reduce the effectiveness of disinfection, necessitate bypass or retrieval procedures, prolong chair-time and ultimately threaten the long-term prognosis of the tooth. By identifying irrigation protocols that either preserve or weaken instrument integrity, our results will help clinicians balance antibacterial efficacy against mechanical safety.

## 2. Materials and Methods

### 2.1. Focused Question

This systematic review was designed in accordance with the PICO framework, addressing the following question: In nickel–titanium rotary instruments used in endodontic procedures (Population), does exposure to endodontic irrigants (Intervention) result in altered cyclic fatigue resistance (Outcome) compared to instruments not exposed to irrigants or exposed to control solutions (Comparison)?

### 2.2. Protocol

**Figure 2:**
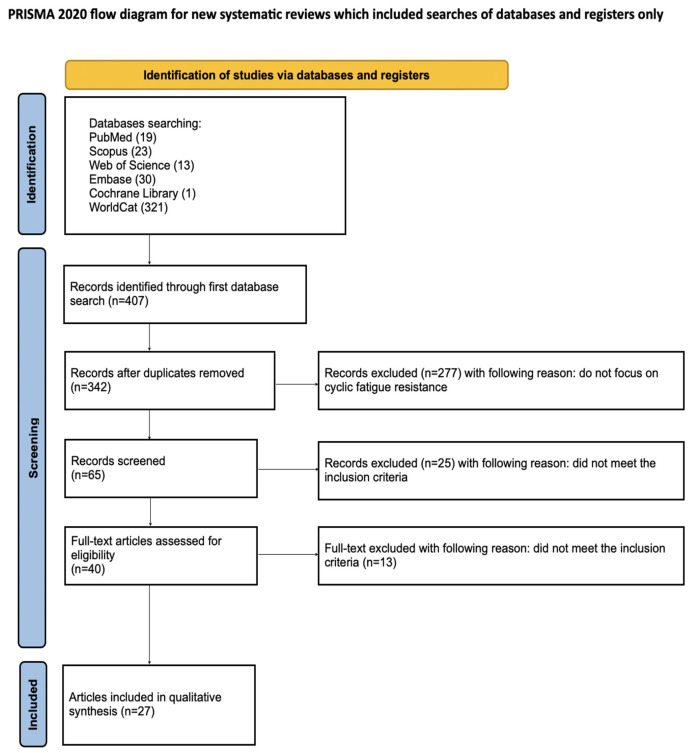
The PRISMA 2020 flow diagram [15].

The article selection process for the systematic review was carefully outlined using the PRISMA flow diagram (Figure 2). The systematic review was registered with the Open Science Framework under the following link: osf.io/5nb8a (accessed on 24 July 2025) [14].

### 2.3. Eligibility Criteria

Studies were considered acceptable for inclusion in the review if they met the following criteria [16,17,18,19,20,21]:

- Investigation of cyclic fatigue resistance of NiTi rotary instruments depending on irrigant solutions
- Changes in specified surfaces evaluated using optical microscope and/or Scanning Electron Microscope (SEM) and/or profilometers and/or Micro-Computed Tomography (Micro-CT);
- In vitro studies;
- Studies in English;
- Full-text articles;

The exclusion criteria the reviewers agreed upon were as follows:

- Not an investigation of irrigant solutions on cyclic fatigue resistance
- Non-English papers;
- Clinical reports;
- Opinions;
- Editorial papers;
- Review articles;
- No full-text accessible;
- Duplicated publications.

No restrictions were applied with regard to the year of publication

### 2.4. Information Sources, Search Strategy, and Study Selection

A comprehensive literature search was conducted in June 2025 across six electronic databases: PubMed, Scopus, Web of Science, Embase, Cochrane Library, and WorldCat. The search strategy combined controlled vocabulary (e.g., MeSH/Emtree terms where applicable) and free-text keywords with Boolean operators. No restrictions were applied regarding the year of publication. Filters were set to include only full-text, peer-reviewed articles published in English. Only studies fulfilling the predefined eligibility criteria were considered for inclusion.

The general search string applied was:

(“Nickel-Titanium” OR “NiTi files”) AND (“Sodium Hypochlorite” OR NaOCl OR EDTA OR “irrigation solutions”) AND (“Fatigue” OR “cyclic fatigue resistance” OR “instrument fracture”) AND (“Dental Instruments” OR “rotary endodontic instruments”)

Database-specific adaptations were as follows:

PubMed: (“Nickel-Titanium”[Mesh] OR “NiTi files”) AND (“Sodium Hypochlorite”[Mesh] OR NaOCl OR EDTA OR “irrigation solutions”) AND (“Fatigue”[Mesh] OR “cyclic fatigue resistance” OR “instrument fracture”) AND (“Dental Instruments”[Mesh] OR “rotary endodontic instruments”)

Scopus: TITLE-ABS-KEY(“Nickel-Titanium” OR “NiTi files”) AND TITLE-ABS-KEY(“Sodium Hypochlorite” OR NaOCl OR EDTA OR “irrigation solutions”) AND TITLE-ABS-KEY(“Fatigue” OR “cyclic fatigue resistance” OR “instrument fracture”) AND TITLE-ABS-KEY(“Dental Instruments” OR “rotary endodontic instruments”)

Web of Science (WoS):

TS = (“Nickel-Titanium” OR “NiTi files”) AND TS = (“Sodium Hypochlorite” OR NaOCl OR EDTA OR “irrigation solutions”) AND TS = (“Fatigue” OR “cyclic fatigue resistance” OR “instrument fracture”) AND TS = (“Dental Instruments” OR “rotary endodontic instruments”)

Embase: (‘nickel titanium’/exp OR ‘niti files’) AND (‘sodium hypochlorite’/exp OR naocl OR edta OR ‘irrigation solutions’) AND (‘fatigue’/exp OR ‘cyclic fatigue resistance’ OR ‘instrument fracture’) AND (‘dental instruments’/exp OR ‘rotary endodontic instruments’)

Cochrane Library: (“Nickel-Titanium” OR “NiTi files”) AND (“Sodium Hypochlorite” OR NaOCl OR EDTA OR “irrigation solutions”) AND (Fatigue OR “cyclic fatigue resistance” OR “instrument fracture”) AND (“Dental Instruments” OR “rotary endodontic instruments”)

WorldCat: (“Nickel-Titanium” OR “NiTi files”) AND (“Sodium Hypochlorite” OR NaOCl OR EDTA OR “irrigation solutions”) AND (Fatigue OR “cyclic fatigue resistance” OR “instrument fracture”) AND (“Dental Instruments” OR “rotary endodontic instruments”)

### 2.5. Data Collection Process and Data Items

Four reviewers (B.K., A.K., M.L., and Z.N.) independently screened and selected studies that met the inclusion criteria. For each eligible article, data were extracted on the first author’s name, year of publication, study design, article title, specifications of the nickel–titanium rotary instruments used, and their cyclic fatigue resistance in relation to various irrigation solutions. All extracted data were systematically recorded in a standardized Excel spreadsheet.

### 2.6. Risk of Bias and Quality Assessment

During the preliminary phase of study selection, each reviewer independently assessed the titles and abstracts to reduce the risk of selection bias. Inter-reviewer agreement was measured using Cohen’s kappa statistic. Any disagreements regarding the inclusion or exclusion of studies were resolved through group discussion and consensus among the reviewers.

### 2.7. Quality Assessment

Two blinded reviewers (J.M. and M.D.) independently examined the methodological quality of each selected study utilizing the Joanna Briggs Institute (JBI) assessment tool for quasi-experimental designs (non-randomized experimental studies). This evaluation instrument contains nine specific criteria formulated to assess the methodological quality of such investigations.

- Is it clear in the study what is the ‘cause’ and what is the ‘effect’?
- Were the participants included in any similar comparisons?
- Were the participants included in any comparisons receiving similar treatment/care, other than the exposure or intervention of interest?
- Was there a control group?
- Were there multiple measurements of the outcome both before and after the intervention/exposure?
- Was a follow-up completed, and if not, were differences between groups in terms of their follow-up adequately described and analyzed?
- Were the outcomes of participants included in any comparisons measured in the same way?
- Were the outcomes measured in a reliable way?
- Was an appropriate statistical analysis used?

Each item on the checklist was rated as “yes,” “no,” “unclear,” or “not applicable.” When reviewers provided conflicting responses, disagreements were addressed through discussion until a consensus was reached. Inter-rater reliability was assessed using Cohen’s kappa statistic, calculated with MedCalc software (version 23.1.7; MedCalc Software Ltd., Brussels, Belgium). The resulting kappa value of 0.86 (*p* < 0.001) indicated a high level of agreement, demonstrating near-perfect consistency among the reviewers.

## 3. Results

### 3.1. Study Selection

The initial search of PubMed, Scopus, WoS, Embase, Cochrane Library and WorldCat databases yielded 407 potentially relevant articles. After removing duplicates, 342 articles remained, and studies not related to irrigants solutions effects on cycling fatigue resistance were excluded. The remaining 65 articles were screened. After an initial search of titles and abstracts, 25 articles that did not meet the inclusion criteria were excluded. Of the remaining 40 studies, 13 did not meet the inclusion criteria after full text analysis. Ultimately, a total of 27 articles were included in the qualitative synthesis of this review. The considerable heterogeneity of the included studies prevented the conduct of a meta-analysis.

### 3.2. General Characteristics of the Included Studies

The included studies displayed considerable heterogeneity in methodology, with no single standardized testing protocol. A total of eight studies assessed only one NiTi file system [15,22,23,24,25,26,27], while nineteen studies investigated multiple systems [28,29,30,31,32,33,34,35,36,37,38,39,40,41,42,43,44,45,46]. In most studies, the instrument sizes were consistent across groups, with only three exceptions where different sizes were used [22,26,36]. Variability in taper was more common, with discrepancies noted in fourteen studies [15,22,28,29,30,31,32,33,36,40,41,42,43,44]. Twelve studies evaluated instruments made from conventional austenitic NiTi alloy [12,14,15,18,21,23,24,25,26,28,32,34], whereas nineteen involved heat-treated NiTi instruments [25,27,29,30,31,32,33,34,35,37,39,40,41,42,43,44,45,46,47]. Both alloy types were assessed in seven studies [30,33,35,37,40,41,45]. Regarding testing models, the most frequently used canal curvature was 60° [24,25,27,32,33,34,35,36,37,38,40,41,42,43,44,45,46], followed by 45° [22,23,29,30,39], 75° [31], and 90° [26,47]. Only one study evaluated more than one curvature configuration [15] (see Table 1).

Sodium hypochlorite (NaOCl) was the most commonly used irrigant, featured in 26 studies [15,22,23,24,25,26,27,28,29,30,31,32,33,34,35,36,37,38,39,40,41,42,43,44,45,47]. In most cases, a single concentration was used, except in two studies where multiple concentrations were tested [27,43]. The most frequent concentration was 5–5.25% (19 studies) [22,23,24,25,26,27,29,31,33,34,35,36,38,39,40,41,42,43,46], followed by 2.5% [27,32,37,43], 1.2% [15,28], 3% [30,44], and 6% [44,47]. The irrigant temperature varied across studies. Most used room temperature, although body temperature (37 °C) was the most common in those that used heated irrigants [24,27,30,32,33,38,40,41,42,43,46]. Six studies evaluated more than one temperature setting [25,27,35,41,43,46]. NaOCl was compared with distilled or deionized water in eight studies [15,22,27,35,40,41,43,44], with EDTA in three [39,43,44], chlorhexidine in one [47], and saline in one [26]. One study assessed only water, with fatigue testing performed at four different temperatures (3 °C, 22 °C, 37 °C, 60 °C) [46]. To facilitate comparability across future studies, these findings highlight the need for standardized protocols. Specifically, NaOCl should be tested at clinically relevant concentrations (2.5–5.25%) and maintained at 37 °C to simulate intraoral conditions. EDTA (17%) and CHX (2%) should also be evaluated under the same temperature setting, while distilled water, saline, and other solutions should continue to serve as controls.

All studies assessed the impact of irrigants on cyclic fatigue resistance. In over half of the studies, instruments were immersed in irrigants prior to testing [23,24,26,29,30,31,32,33,34,36,37,38,39,41,45], while in fewer cases, immersion occurred during testing [15,25,27,35,42,43,44,46,47]. Only one study combined pre- and intra-test immersion [22].

Five studies incorporated autoclave sterilization as an additional experimental factor prior to fatigue testing [26,31,33,34,37].

**Figure 3:**
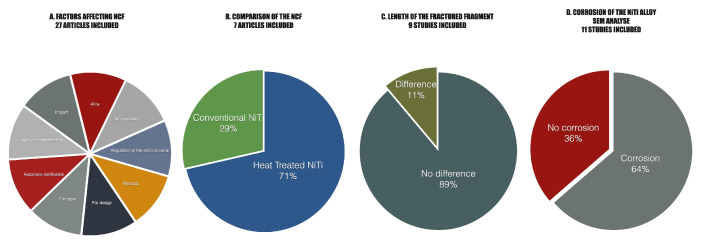
Summary of findings from included studies regarding cyclic fatigue resistance of NiTi instruments. (**A**) comparison of heat-treated vs. conventional NiTi in terms of number of cycles to failure (NCF); (**B**) factors influencing NCF identified across studies; (**C**) reported differences in fractured fragment length; and (**D**) presence of corrosion on fractured surfaces based on SEM analysis.

Seventeen studies employed scanning electron microscopy (SEM) to evaluate post-fracture surface features, including corrosion and microcrack formation [15,22,23,24,27,28,30,33,34,35,39,40,41,42,43,44,47]. Among these, eleven studies specifically assessed corrosion, with 64% confirming signs of corrosion and 36% reporting no corrosion of the NiTi alloy (see Figure 3).

### 3.3. Main Study Outcomes

All included studies evaluated cyclic fatigue resistance, either expressed as the number of cycles to fracture (NCF) or as time to fracture, which can be converted to NCF based on the rotational speed (RPM) of the instrument. In addition, a substantial proportion of studies assessed post-fracture surface characteristics using scanning electron microscopy (SEM) [15,22,23,24,27,28,30,33,34,35,39,40,41,42,43,44,47]. (see Table 2)

#### 3.3.1. Number of Cycles to Fracture (NCF)

The number of cycles to fracture was the primary outcome measure in all included studies. This parameter was evaluated under diverse experimental conditions, including different file systems, irrigant types and concentrations, exposure temperatures, and durations of immersion, as detailed in Section 3.2.

Among the 27 studies, eleven investigated instruments made from conventional austenitic NiTi alloy [15,26,30,33,35,36,37,38,40,41,45], while nineteen studies used heat-treated NiTi systems such as Blue Wire (VDW), Gold Wire (Dentsply), CM Wire (Coltene), or EDM Wire (Coltene) [25,27,29,30,31,32,33,34,35,37,39,40,41,42,43,44,45,46,47]. Seven studies directly compared both alloy types within the same experimental design [30,33,35,37,40,41,45]. In most cases, the differences between conventional and CM wire instruments were highly significant and readily observable. Palma et al. [30] reported that the mean NCF after 5 min of immersion was 1172 for ProTaper Next and 5508.33 for HyFlex EDM. Similarly, Huang et al. [35] compared K3 and Vortex files under different conditions; cyclic fatigue testing in 60 °C sodium hypochlorite revealed NCF values of 284.17 and 609.72, respectively. These findings underline the superior fatigue resistance of modern heat-treated alloys, which may translate into reduced risk of unexpected file fracture and greater safety in challenging clinical situations.

In most of these comparative studies, heat-treated alloys exhibited significantly greater resistance to cyclic fatigue, with higher NCF values reported for files such as Reciproc Blue, WaveOne Gold, and HyFlex EDM compared to conventional counterparts [30,35,40,41,45]. This difference is typically attributed to enhanced flexibility and improved metallurgical properties imparted by proprietary thermomechanical processing. However, one study by Kermeoğlu et al. found that a conventional reciprocating instrument made from M-Wire outperformed a heat-treated file, suggesting that other factors such as motion type (reciprocation vs. continuous rotation) and cross-sectional design may influence fatigue resistance [33].

Statistical significance in NCF reduction due to irrigant exposure—especially immersion in sodium hypochlorite (NaOCl) or elevated temperature—was reported in nineteen studies [15,24,27,29,30,31,32,33,34,35,36,38,40,41,42,43,44,46,47]. These findings support the hypothesis that aggressive or heated irrigants may compromise the structural integrity of NiTi alloys by initiating surface corrosion or microstructural changes.

Conversely, ten studies found no significant difference in NCF following irrigant exposure [23,25,26,29,33,34,37,38,39,45]. Notably, Javadi et al. demonstrated that the effect of NaOCl immersion was file-specific—reducing the NCF of the M3 file but not affecting the SP1 file, despite both being manufactured from heat-treated alloy [29]. Similarly, Pedullà et al. observed no impact of NaOCl on fatigue resistance but reported superior performance of Reciproc over WaveOne, indicating that instrument design may outweigh irrigant effects in certain conditions [38].

The results of recent studies corroborate the enhanced properties of heat-treated NiTi alloys. Thermal processing induces the formation of a titanium oxide layer on the wire surface. This protective layer increases surface hardness, thereby reducing susceptibility to degradation by sodium hypochlorite. Furthermore, the improved surface homogeneity and smoothness minimize the initiation of corrosion pits, contributing to greater structural integrity and durability of the instruments.

#### 3.3.2. Fracture Fragment Length

Fracture fragment length was evaluated in nine studies as a secondary outcome, intended to assess whether irrigant exposure or testing conditions affected the location and consistency of file failure. Eight studies concluded that irrigant type, concentration, or exposure temperature had no significant influence on the length of the fractured fragment [25,27,28,37,40,44,47]. These results suggest that, although irrigants may reduce the instrument’s fatigue life, they do not appear to influence the site of fracture along the instrument shaft.

Only one study, conducted by Kermeoğlu et al., reported a difference in fragment length, which was attributed to multiple autoclave sterilization cycles rather than irrigant exposure [33]. The study noted shorter fragment lengths in files subjected to repeated sterilization, indicating that thermal cycling may weaken certain portions of the instrument.

#### 3.3.3. Scanning Electron Microscopy (SEM) Analysis

Scanning Electron Microscopy (SEM) was employed in 17 studies to assess surface degradation, presence of corrosion, and fracture morphology in NiTi files following irrigant exposure [15,22,23,24,27,28,30,33,34,35,39,40,41,42,43,44,47]. In all cases, sodium hypochlorite was used as the test irrigant, either alone or in comparison with other solutions.

More than half of the SEM-based studies confirmed the presence of corrosion or pitting on the instrument surface following immersion in NaOCl [15,24,27,28,39,41,42]. These surface alterations are considered precursors to crack initiation and premature failure under cyclic loading. The studies typically described features such as irregular corrosion pits, intergranular attack, or local erosion, which weaken the passive oxide layer on the NiTi surface and increase susceptibility to fatigue. Alfawaz et al. [27] indicated that cracks often initiate at the cutting edge and may represent the starting point for complete file fracture, a finding later confirmed by Cheung et al. [15]. Moreover, Cheung reported that multiple crack origins can be present within a single instrument [15]. Several studies have also emphasized that immersion in sodium hypochlorite may induce pitting through the leaching of nickel from the instrument surface, although this phenomenon appears to occur less frequently than microcrack formation [15,24,28,42].

In contrast, four studies found no visible signs of corrosion following NaOCl exposure, suggesting that short-term or low-temperature immersion may be insufficient to initiate surface degradation [22,23,35,40]. Differences in alloy composition, surface finish, and protective coatings may also account for the variability in corrosion response.

Eleven studies that utilized SEM identified consistent fracture patterns in failed instruments, including features indicative of ductile failure (e.g., dimples and microvoid coalescence) and fatigue striations, often located near the initiation site [15,27,28,33,34,35,40,41,42,43,44]. These findings corroborate the mechanical fatigue nature of the failure process and highlight the complex interplay between material properties, irrigant chemistry, and cyclic loading.

**Table 2:** Detailed characteristics of included studies.

Author	Instrument Type, Alloy	Irrigant (Type + Conc. + Temp. + Exposure)	Parameters of Simulated Canal	Rotation, Speed	Fatigue Test Method	Fatigue Resistance Results	Surface Analysis (SEM)
de Castro Martins [22]	Total files = 80 Type: ProFile Sizes 20, 25, 30 with 0.04 taper Size 20 with 0.06 taper Alloy: NiTi	5.25% NaOCl deionized water Temp.: Not specified Exposure: EG1: 24 h immersion in NaOCl EG2: Used deionized water for shaping canals EG3: Used NaOCl for shaping canals	Artificial canals—AISI H13 tool steel, 45° angle of curvature and 5 mm radius The area of max. tensile strain amplitude at about 4 mm from the tip of the instrument	Constant speed of 250 rpm, -low speed, -low torque.	Rotating bending fatigue test Conducted using a custom test bench with artificial canals	Fracture location: typically ~4 mm from the tip. CG: NCF –#20/0.04: ≈1000 –#25/0.04: ≈800 –#30/0.04: ≈650 –#20/0.06: ≈700 EG1: NCF –#20/0.04: ≈950 –#25/0.04: ≈750 –#30/0.04: ≈600 –#20/0.06: ≈680 EG2: NCF –#20/0.04: ≈600 –#25/0.04: ≈500 –#30/0.04: ≈450 #20/0.06: ≈400 EG3: –#20/0.04: ≈600 –#25/0.04: ≈500 –#30/0.04: ≈450 –#20/0.06: ≈420 EG2 and EG3 had significantly lower NCF than CG and EG1. EG1 showed no significant change from control. EG3 showed higher fatigue resistance than EG2, but not statistically significant	EG1: No signs of corrosion or surface alteration EG2 & EG3 (post use): Signs of fretting, scratched surfaces, metal flash loss but no corrosion visible under SEM. Presence of microcracks between 2.2 mm and 3.8 mm from tip. SEM of fractured surfaces showed fatigue striations and ductile fracture characteristics
Cheung [28]	Total files = 179 Type: ProFile, K3, HERO, Shaper, FlexMaster Size 25, 0.04 and 0.06 tapers Alloy: NiTi	1.2% NaOCl Temp.: 23 °C ± 2 °C Exposure: Continuous immersion during the entire fatigue test (i.e., real-time exposure during rotation)	Custom-made device mimicking rotational instruments were pre-bent to a curvature (radius Rc measured from photo) Noexact canal length or curvature angle specified—just that curvatures varied and were controlled	-250 rpm -maximum torque	The Low-Cycle Fatigue test (LFC)—rotational-bending low cycle fatigue test Nf < 2000 cycles The test focused on the strain-life relationship, calculating surface strain amplitude using the instrument’s curvature and fracture cross-section diameter. This follows the Coffin–Manson equation, which is standard for LCF evaluation.	ProFile (PF) Number of samples: 30 Y-intercept: 2.2220 Fatigue-ductility exponent (c ± SE): −0.612 ± 0.070 Coefficient of determination (R²): 0.732 K3 Number of samples: 28 Y-intercept: 2.0577 Fatigue-ductility exponent (c ± SE): −0.583 ± 0.094 Coefficient of determination (R²): 0.594 HERO Shaper (HE) Number of samples: 49 Y-intercept: 2.0680 Fatigue-ductility exponent (c ± SE): −0.568 ± 0.049 Coefficient of determination (R²): 0.745 FlexMaster (FM) Number of samples: 29 Y-intercept: 2.2519 Fatigue-ductility exponent (c ± SE): −0.648 ± 0.141 Coefficient of determination (R²): 0.439	SEM was used Corrosion pits were found in ~13% of all samples across all brands. These pits were usually located at or near the vertex of the cross-section. When present, pits were always the site of crack initiation. PF: 14% with pits K3: 13% HE: 13% FM: 13% Multiple crack origins were more common in some brands (e.g., K3: 48% of samples). Fractographic analysis showed that cracks initiated in high-strain areas and then grew inward.
Ormiga Galvão Barbosa [23]	Total files= 32 Type: K3 Size 25, taper 0.06 Alloy: NiTi	5.25% NaOCl Temp.: not specified Exposure: immersion of files in 400 mL of 5.25% NaOCl for up to 8 h	Material: Small glass tube used to simulate canal curvature Angle: 45 degrees Curvature Radius: 5 mm Location of Curvature: Between 3 and 7 mm from the tip of the file	Flexural fatigue tests: 300 rpm Torsional resistance tests: 1 rpm clockwise	Flexural fatigue test: Files rotated in a simulated canal (glass tube with curvature) until fracture, monitored by electrical resistance changes Torsional resistance: Files were clamped and twisted until fracture using a torque meter, torque oscillated within the range of 2.5 to 4.5 Ncm.	Files as Received -Flexural Fatigue Test: Number of cycles: 7993.13 ± 2469.26 -Torsional Resistance Test: Angle (degrees): 481.50 ± 54.59 Torque (N·cm): 3.80 ± 0.59 Files Exposed to NaClO -Flexural Fatigue Test: Number of cycles: 8235.63 ± 1652.55 -Torsional Resistance Test: Angle (degrees): 477.75 ± 103.87 Torque (N·cm): 3.73 ± 0.35	No evidence of localized corrosion or surface changes after exposure to NaClO. SEM images showed similar morphology between new and NaClO-exposed files
Berutti [24]	Total files = 120 Type: ProTaper (F2) Alloy: NiTi	5% NaOCl Temp.: 50 °C Exposure Time: 5 min (EG1—20 mm, EG2—whole files)	Material: Stainless steel artificial canal Curvature: 60° Radius of Curvature: 10 mm Constant air cooling was applied to avoid overheating	Constant speed 300 rpm	Cyclic fatigue test using a motorized endodontic instrument in a stainless steel simulated canal. Instruments rotated until fracture; time to fracture was recorded.	Group 1 (control): Mean fracture time = 142.47 s Group 2 (20 mm immersed): Mean = 136.50 s Group 3 (fully immersed): Mean = 105.15 s Group 3 had significantly lower resistance (*p* < 0.001). Some instruments fractured after only 7 s. Groups 1 and 2 were not significantly different (*p* = 0.42)	SEM revealed corrosion damage in Group 3: localized pitting and cracks, especially near fracture areas. EDS spectra showed nickel and titanium in non-corroded zones; in corroded areas, oxides of Ni and Ti were present along with calcium carbonate (from tap water). Fractures showed intergranular propagation linked to corrosion sites.
Javadi [29]	Total files= 90 Type: M3 Pro Gold, SP1 (V-Taper Gold). M3 size 25, taper 0.06 SP1 size F2 Alloy: M3 Pro Gold: Gold-treated CM wire (Controlled Memory alloy), composed of martensite and austenite phases at room temperature. SP1: Manufactured using H-wire technology, exact alloy composition is not disclosed.	5% NaOCl Deconex Temp.: Room temperature (~25 °C) Exposure: 5 min	Material: Stainless steel groove in chromium-cobalt block Shape: Corresponded to size #25/0.06 gutta-percha Curvature: 45° angle, 5 mm radius of curvature	SP1: 350 rpm, torque 2 N·cm M3 Pro Gold: 350 rpm, torque 3 N·cm	Cyclic fatigue test performed using a custom- made device. Files were rotated in an artificial canal until fracture. The setup included a motorized handpiece mounted on a fixed base. Tempered glass cover was used for visual monitoring. Lubricant spray (Hi-Clean) used to reduce friction.	NCF (M3)—Deconex: 79.67 ± 8.2 NCF (M3)—5% NaOCl: 72.93 ± 6.92 NCF (M3)—No immersion: 111.6 ± 7.02 NCF (M3)—Total: 88.07 ± 18.53 NCF (SP1)—Deconex: 158.53 ± 8.14 NCF (SP1)—5% NaOCl: 120.67 ± 19.7 NCF (SP1)—No immersion: 118 ± 19.08 NCF (SP1)—Total: 132.4 ± 23.46 NCF (Total)—Deconex: 119.1 ± 40.9 NCF (Total)—5% NaOCl: 96.8 ± 28.28 NCF (Total)—No immersion: 114.8 ± 10.82 NCF (Total)—Total: 110.23 ± 30.63 Highest resistance: SP1 in Deconex (158.53 ± 8.14) Lowest resistance: M3 in NaOCl (72.93 ± 6.92) SP1 files had significantly higher resistance than M3 files overall (*p* < 0.001). NaOCl immersion reduced fatigue resistance, especially for M3. Deconex either maintained or increased resistance, especially in SP1.	None
Palma [30]	Total files= 90 Type: ProTaper Next (PTN), Hyflex CM (CM), Hyflex EDM (EDM) Size 25, taper 0.06 (PTN, CM) Size 25, variable taper (EDM) Alloy: NiTi	3% NaOCl Temp.: 37 °C Exposure: 1 min or 5 min of dynamic immersion (rotating in a glass container)	Cyclic fatigue testing inside a curved canal milled from stainless-steel blocks Length: 16 mm Curvature: 45° angle, Radius: 5 mm Width: 1.4 mm coronally, 0.5 mm apically The axial movement of the canal = 3.5 mm amplitude at ⅓ Hz	PTN: 300 rpm, torque 2.0 N·cm CM and EDM: 500 rpm, torque 2.5 N·cm	Dynamic cyclic fatigue model with axial movement. Performed in an artificial canal heated to 37 °C Number of cycles to fracture recorded by software	NCF: PTN: -Without immersion: 1237.50 ± 177.24 -After 1 min in NaOCl: 1193.00 ± 184.69 (3.6% reduction) -After 5 min in NaOCl: 1172.00 ± 186.31 (5.3% reduction) -*p* value: < 0.01 CM: -Without immersion: 2155.60 ± 372.25 -After 1 min in NaOCl: 1833.32 ± 346.33 (15% reduction) -After 5 min in NaOCl: 1859.17 ± 307.32 (14% reduction) -*p* value: < 0.01 EDM: -Without immersion: 6028.33 ± 1012.20 -After 1 min in NaOCl: 5182.32 ± 829.46 (14% reduction) -After 5 min in NaOCl: 5508.33 ± 480.89 (9% reduction) -*p* value: < 0.01 NaOCl reduced fatigue resistance in all systems, especially in CM and EDM.	SEM analysis was performed on two randomly selected samples from each group to examine: Fracture surfaces. All files showed features typical of cyclic fatigue: -Initiation zone, -Crack propagation zone, -Final fracture zone with visible dimples. Surface condition—After immersion in NaOCl (especially 5 min), corrosion was observed in the form of: -Craters -Pitting defects More evident on EDM instruments due to their rougher surfaces.
Devi Priya [31]	320 files TruNatomy (NiTi wire that is used to manufacture most generic files, followed by a special heat treatment), Hyflex CM (controlled memory wire with a lower Nickel content), Hyflex EDM (controlled memory wire with electric discharge machining (EDM), EdgeFile X3 (Fire-Wire NiTi alloy) Size 25.06 20 files per subgroup	Subgroup1. Control group S2. NaOCl 5.25% for 3 min S3. Autoclave 3 cycles 121 °C S4. NaOCl 5.25% for 3 min and Autoclave 3 cycles 121 °C	75° angle of curvature, a 5 mm radius of curvature to the center, overall length of 17 mm. Built with Schneider’s technique.	Continuous rotation 500 rpm	Fatigue test was performed after the immersion and autoclave. The tool allowed the instrument to revolve freely and under steady pressure inside the man-made canal. Till a fracture developed, all instruments were turned. The mechanical parts were lubricated with a synthetic oil (Turbo X Spray Plus (NSK, Tokyo, Japan))	NCF TruNatomy S1. 271.11 *±* 56.22 S2. 277.33 *±* 34.22 S3. 185.00 *±* 99.00 S4. 366.33 *±* 74.22 HyflexCM S1. 433.99 *±* 125.22 S2. 811.00 251.00 S3. 666.22 *±* 266.00 S4. 700.22 *±* 231.22 HyflexEDM S1. 768.88 *±* 109.22 S2. 721.33 *±* 93.22 S3. 725.00 *±* 86.00 S4. 963.00 *±* 221.96 EdgefileX3 S1. 803.28 *±* 171.22 S2. 1222.31 *±* 266.22 S3. 1355.00 *±* 581.00 S4. 1495.00 *±* 394.22	None
Mousavi [25]	45 files, 15 per subgroup ProTaper Gold S1	NaOCl 5.25% at 22 °C in group 1, at 4 °C in group 2, and at 37 °C in group 3. ±1 °C	Stainless-steel metal block, a curvature angle of 60°, a curvature radius of 5 mm and a length of 25 mm	Rotated at 300 rpm	The block was fixed inside a recipient that was filled with 5.25% sodium hypochlorite. The block and handpiece were fixed in place with a clamp.	NCF Group 1 1248.50000 ±299.17526 4.42 ± 0.45 Group 2 1076.50000 ±190.93411 4.98 ± 0.56 Group 3 1119.50000 ±117.81174 4.48 ± 0.71	None
Tanomaru [47]	30 files, 10 per subgroup XP-Endo Finisher	Group 1: XPF instruments using 2% chlorhexidine gel as an irrigation solution; Group 2: XPF using 6% sodium hypochlo- rite as an irrigation solution; Group 3 (control group): XPF instruments using lubricating oil (WD-40, Milton Keynes, UK).	Artificial groove measuring 1.5 mm wide, 20 mm long and 3.5 mm deep with a straight cervical segment measuring 14.29 mm, with an arc length of curvature of 4.71 mm, with a radius of curvature of 3 mm angle 90 degree, and ending in a linear apical segment of 1 mm simulating root canals	The speed is 900 RPM and torque 1N·cm.	The irrigant was taken to the artificial canal with Navi tip plastic needles, with a 21 mm long flexible tip penetrating 1 mm into the cervical region of the simulated canal with the needle tip without touching the moving file, maintaining the canal filled with the solution. The files were inserted in the simulated canals until the length of 20 mm, using a stop to register this length.	Time and NCF CHX T-4.99 (1.62) NCF 4558.5 (1495.33) NaOCl T-0.92 (0.91) NCF 1014.00 (795.34) Oil T-0.67 (0.41) NCF 832.50 (354.42) Fragment length CHX 3.90 (0.188) NaOCl 4.05 (0.236) LO 3.82 (0.187)	Analyzed under SEM. Absence of plastic deformation in the helical shaft of all instruments
Abuhulaibah [32]	90 files 15 per subgroup One Curve (OC) iso25 (heat-treated C-wire technology ) and ProTaper Gold (PTG) F2	S1. No immersion (control), S2. 1 min immersion, S3. 5 min immersion in 2.5%NaOCl at 37 °C.	25 mm length, with a single curvature of 60° and a 5 mm radius that was engraved into the block to a depth of 1.5 mm.	Clockwise rotation with individual manufacturer’s recommended speed for each system.	Immersion in sodium hypo was performed before the fatigue test. Test of the files was conducted in a water bath machine, filled with water maintained at 37 °C. A lubrication spray (Pegasus Hand Piece Lubrication Spray, UK) was introduced within the canal space to reduce frictional heat.	NCF PTG-control 752.00 ± 165.37 PTG-1 min NaOCl 651.00 ± 165.64 PTG-5 min NaOCl 490.33 ± 120.65 OC-control 1325.00 ± 334.0 OC-1 min NaOCl 1038.33 ± 204.12 OC-5-min NaOCl 962.33 ± 145.80	None
Svec [26]	75 files, 25 per group, 5 per subroup ProFile 0.04 taper rotary files 25 mm long in ISO sizes 25, 30, and 35	Group A control (no rotation, no autoclave, no sodium hypo) Group B Saline, no autoclave Group C Saline, autoclave between cycycles Group D Sodium hypo 5.25%, no autoclave Group E Sodium Hypo 5.25% autoclave between cycles	The tube had a 90° curve with a 5 mm radius of curvature.	150 rpm	Groups B, C, D, and E were exposed to cyclic fatigue three times for 30 s each time. The files were used in an axial motion with a range of ~6 mm. Group C and E was autoclaved 132C, 30 min between each cycle Torsional moment (N-cm) and angular deflection (degrees) at failure were measured on the Torsiometer/Memocouple	Torsional moment of rotary files Size 25 A 0.78 (0.04) B 0.84 (0.06) C 0.84 (0.07) D 0.81 (0.04) E 0.83 (0.08) Size 30 A 1.06 (0.10) B 1.15 (0.09) C 1.13 (0.10) D 1.10 (0.17 E 1.18 (0.05) Size 35 A 1.47 (0.15) B 1.30 (0.10) C 1.30 (0.13) D 1.38 (0.08) E 1.30 (0.19) Angular deflection of rotary files Size 25 A 500 (30) B 620 (60) C 690 (120) D 580 (70) E 570 (90) Size 30 A 480 (40) B 680 (70) C 620 (110) D 640 (120) E 620 (110) Size 35 A 810 (150) B 790 (60) C 820 (70) D 720 (80) E 610 (120)	None
Kermeoglu [33]	210 files, 70 per type, 10 per subgroup (iso 25) Reciproc R25 file, ProTaper Universal F2 file and Wave One Gold Primary file	Subgroup 1 (control): no immersion Subgroup 2: immersion in 5.25% NaOCl at a temperature of 37 °C was performed for 5 min Subgroup 3: immersion in Irritrol at a temperature of 37 °C was carried out for 5 min. Subgroup 4: immersion in 5.25% NaOCl at a tempera- ture of 37 °C for 5 min with one-cycle autoclave steril- isation was performed. Subgroup 5: the procedure of subgroup 4 was repeated three times. Subgroup 6: immersion in Irritrol at a temperature of 37 °C for 5 min with one-cycle autoclave sterilization was performed. Subgroup 7: the procedure of subgroup 6 was repeated three times.	A stainless steel artificial canal was produced as a result of reproducing the size and taper of the instrument with an angle of curvature of 60°, a radius of curvature of 5 mm and the curvature center-positioned 5 mm from the artificial canal’s tip	Reciproc ALL PTU instruments were utilized with the identical endodontic motor at 500 rpm and the highest torque level WaveOne ALL	Immersion and autoclave was performed before the fatigue test. The dynamic cyclic fatigue test was conducted on the chosen instruments by utilizing a custom-made cyclic fatigue testing device. For the fatigue test in SS artificial block there was an application of synthetic oil (Super Oil; Singer Co Ltd., Elizabethport, NJ, USA) All instruments were rotated in a freeway within the artificial canal until occurring a fracture.	TF ProTaper Control: 29.94 ± 5.62 Subgroup 2: 22.65 ± 9.94 Subgroup 3: 28.31 ± 4.95 Subgroup 4: 19.29 ± 4.79 Subgroup 5: 14.09 ± 4.95 Subgroup 6: 18.75 ± 3.44 Subgroup 7: 13.22 ± 4.08 Total: 20.89 ± 8.16 WaveOne Gold Control: 51.38 ± 11.81 Subgroup 2: 47.90 ± 3.02 Subgroup 3: 47.82 ± 5.23 Subgroup 4: 45.15 ± 12.20 Subgroup 5: 42.65 ± 15.28 Subgroup 6: 45.54 ± 17.60 Subgroup 7: 40.00 ± 15.68 Total: 45.78 ± 12.51 Reciproc Control: 126.90 ± 20.50 Subgroup 2: 122.79 ± 21.85 Subgroup 3: 113.60 ± 23.71 Subgroup 4: 112.32 ± 20.68 Subgroup 5: 108.36 ± 30.96 Subgroup 6: 111.62 ± 21.86 Subgroup 7: 93.68 ± 18.61 Total: 112.75 ± 24.04 Total Control: 69.40 ± 44.41 Subgroup 2: 64.45 ± 45.30 Subgroup 3: 63.24 ± 39.59 Subgroup 4: 58.92 ± 42.15 Subgroup 5: 55.04 ± 44.60 Subgroup 6: 58.64 ± 42.68 Subgroup 7: 48.97 ± 36.70 Total: 59.81 ± 42.15 Length ProTaper Control: 3.37 ± 0.36 Subgroup 2: 2.97 ± 0.27 Subgroup 3: 3.04 ± 0.25 Subgroup 4: 2.81 ± 0.36 Subgroup 5: 3.20 ± 0.19 Subgroup 6: 3.19 ± 0.25 Subgroup 7: 3.42 ± 0.32 Total: 3.14 ± 0.35 WaveOne Gold Control: 2.28 ± 0.09 Subgroup 2: 2.31 ± 0.07 Subgroup 3: 2.28 ± 0.12 Subgroup 4: 2.63 ± 0.32 Subgroup 5: 3.18 ± 0.77 Subgroup 6: 3.46 ± 0.67 Subgroup 7: 2.44 ± 0.25 Total: 2.65 ± 0.60 Reciproc Control: 2.17 ± 0.22 Subgroup 2: 2.08 ± 0.15 Subgroup 3: 2.12 ± 0.17 Subgroup 4: 3.17 ± 1.00 Subgroup 5: 3.36 ± 0.93 Subgroup 6: 3.68 ± 0.29 Subgroup 7: 3.85 ± 0.73 Total: 2.92 ± 0.93 Total Control: 2.60 ± 0.60 Subgroup 2: 2.45 ± 0.40 Subgroup 3: 2.48 ± 0.44 Subgroup 4: 2.87 ± 0.66 Subgroup 5: 3.25 ± 0.69 Subgroup 6: 3.44 ± 0.48 Subgroup 7: 3.24 ± 0.76 Total: 2.90 ± 0.69	The SEM images of the fractured surfaces of the Pro- Taper, WOG and Reciproc files after the cyclic fatigue test under 3009 and 30,009 magnifications showed the typical pattern of a cyclic fatigue fracture with a fatigue striation and circular abrasion mark. Similarly to previous studies,
Pedulla [34]	210 files, 15 per subgroup per type (25/0.06) Twisted Files and Hyflex CM	Group 1 (control group) no autoclave, no sodium hypo Groups 2—1 time immersion in sodium hypo 5% for 3 min. Group3—3 times immersion in sodium hypo 5% for 3 min. Groups 4—1 time autoclave Group 5—3 times autoclave Group 6—immersion in sodium hypo and 1 time autoclave Group 7 immersion in NaOCl and 3 times autoclave.	Metal block with a suitable artificial canal with a 60 degree angle of curvature and a 5 mm radius of curvature to the center of the 1.5-mm-wide canal.	Continuous rotation at 500 rpm.	Dynamic immersion (1 or 3 min) was allowed, activating the endodontic instruments with a 500 rpm. Autoclave 134, 17 min. Immersion was before autoclaving	NCF Instrument: Hyflex CM Group 1: n = 15, NCF = 710.5, SD = 71.2 Group 2: n = 15, NCF = 792.6, SD = 55.8 Group 3: n = 15, NCF = 686.4, SD = 63.5 Group 4: n = 15, NCF = 750.0, SD = 51.5 Group 5: n = 15, NCF = 797.5, SD = 65.0 Group 6: n = 15, NCF = 681.4, SD = 42.6 Group 7: n = 15, NCF = 732.2, SD = 74.5 Instrument: Twisted File Group 1: n = 15, NCF = 705.8, SD = 75.8 Group 2: n = 15, NCF = 674.4, SD = 77.6 Group 3: n = 15, NCF = 661.4, SD = 81.5 Group 4: n = 15, NCF = 638.9, SD = 70.2 Group 5: n = 15, NCF = 484.9, SD = 74.4 Group 6: n = 15, NCF = 641.9, SD = 83.7 Group 7: n = 15, NCF = 548.8, SD = 68.2	SEM analyses Clear crack initiation zones and overload fast fracture zones, both typical of cyclic fatigue failure, were evident on all samples. Surface Grooves: -HyFlex CM files exhibited grooves orthogonal (perpendicular) to their longitudinal axis. -Twisted Files (TFs) displayed grooves parallel to the longitudinal axis.
Huang [35]	K3, K3XF, Vortex 12 files per group (size 25/0.04),	Sodium hypochlorite 5.25% and distilled water in 3 temperatures: 2 °C, 37 °C, 60 °C.	Novel artificial ceramic canal with a curvature of 60 degree and a 5 mm radius.	K3 was rotated at 300 rpm, and K3XF and Vortex were allowed to rotate at 500 rpm as recommended by the manufacturer	Fixed in a glass container filled with 300 mL 5.25% NaOCl or distilled water that was placed on a hot plate	K3 -Distilled water 501.54 ± 70.46 at 22 °C, 413.75 ± 46.67 at 37 °C, and 328.75 ± 47.87 at 60 °C. -In 5.25% sodium hypochlorite 433.33 ± 57.98 at 22 °C, 348.33 ± 72.00 at 37 °C, and 284.17 ± 38.54 at 60 °C. K3XF -In distilled water: 914.58 ± 215.30 revolutions at 22 °C, 603.47 ± 112.45 at 37 °C, and 326.00 ± 39.07 at 60 °C. -In 5.25% NaOCl: 884.72 ± 242.96 at 22 °C, 505.56 ± 73.29 at 37 °C, and 293.06 ± 62.45 at 60 °C. Vortex -In distilled water: 1356.94 ± 126.87 revolutions at 22 °C, 1102.78 ± 84.93 at 37 °C, 638.89 ± 64.46 at 60 °C. -In 5.25% NaOCl: 1197.92 ± 64.76 at 22 °C, 1098.61 ± 90.65 at 37 °C, 609.72 ± 77.59 at 60 °C.	Typical fracture patterns were present in all 3 files including 1 or 2 crack initiation areas, the presence of fatigue striations, and a fast fracture zone with dimples. No pitting or crevice corrosion in water or 5.25% NaOCl was present
Topcuoglu [36]	90 files, 30 per brand, 15 per group, ProTaper D3 (size 20, 0.07 taper), D-RaCe DR2 (size 25, 0.04 taper), and Mtwo R2 (size 25, 0.05 taper).	Group 1—no immersion (control group) Group 2 immersion in 5% NaOCl at 37 °C for 5 min. (16 mm of the working part).	60° angle of curvature 5 mm radius of curvature. Measuring method of Schneider. curvature center 5 mm from the instrument tip	Manufacturer’s recommendation (ProTaper D3; 400 rpm, D-RaCe DR2; 600 rpm, Mtwo R2, 280 rpm)	Immersion before the fatigue test, special oil (WD-40 Company, Milton Keynes, UK) was used for lubrication. The diameter of the simulated canal was higher than the instruments allowing free rotation. Instrument rotated freely at a constant pressure	NCF: NaOCl: PT 703.07 ± 212.14 Mtwo 728.33 ± 189.74 D-race 832.67 ± 257.38 No immersion: PT 744.93 ± 283.21 Mtwo 785.07 ± 164.43 D-race—949.87 ± 266.94 Fragment length: NaOCl: PT 4.69 ± 0.2 Mtwo 5.08 ± 0.4 D-race 5.06 ± 0.3 No immersion: PT—4.30 ± 0.3 Mtwo—4.91 ± 0.5 D-race—5.00 ± 0.4	None
Alfawaz [27]	135 files 15 files per group ProTaper Gold F2	Three liquids—distilled water, Sodium hypochlorite 2.5%, Sodium Hypochlorite 5.25% All in three temperatures 25, 37, 60 Celsius degree.	curvature angle of 60 degree, a curvature radius of 5 mm, and a curvature center 5 mm from the instrument tip	Rotation at 300 rpm until fracturing occurred.	The block was fixed inside a recipient that was filled with tested liquids. endodontic rotary motor was mounted on a device that allowed reproducible and fixed positioning	NCF Fragment length At 25 °C, the number of cycles to fracture (NCF) for ProTaper Gold F2 was: 1239.1 ± 388.2 in distilled water, 761.3 ± 270.9 in 2.5% NaOCl 877.8 ± 298.0 in 5.25% NaOCl At 37 °C, the NCF values were: 962.9 ± 276.0 in distilled water, 662.3 ± 227.9 in 2.5% NaOCl, 571.1 ± 172.3 in 5.25% NaOCl. At 60 °C, the NCF values were: 418.8 ± 114.8 in distilled water, 325.9 ± 116.9 in 2.5% NaOCl, 305.6 ± 90.6 in 5.25% NaOCl. The length of the fractured fragments was in the range between 4.1 ± 0.77 and 5.2 ± 0.78 mm.	The fracture surfaces of PTG F2 instruments observed under scanning electron microscopy (SEM) at various temperatures and irrigation conditions exhibited characteristic features of cyclic fatigue. These included one or two crack initiation sites, the presence of fatigue striations, and a fast-fracture zone marked by dimples. Examination of the fracture cross-sections revealed that cracks consistently initiated at the cutting edges, with microscopic dimples evident on the fracture surfaces. Notably, none of the tested instruments showed signs of pitting or crevice corrosion when exposed to water or 5.25% NaOCl, as confirmed by SEM analysis.
Bulem [37]	160 files, 40 per brand, 10 per group—25.06 ProFile FlexMaster Mtwo Twisted File	Group 1—Sodium Hypo 2.5%, 5 min, room temperature Group 2—Sodium Hypo 2.5%, 5 min, room temperature and 1 autoclave cycle 135 °C, 32.5 minutes Group 3—5 full cycles of Group 2 protocol 5× (sodium hypo-autoclave) Group 4—control group—no sodium hypo, no autoclave	60° angle, 2.5 mm radius, curvature beginning 2 mm from the tip of the file	300 rpm for Profile, FlexMasrter, Mtwo 500 rpm for Twisted File	Immersion and autoclave was performed before fatigue test Endodontic motor was fixed, with no movement	ProFile (n = 40): Control: NCF = 396.5 (SD = 77.46), Fragment length = 2.6 mm (SD = 0.4) 5 min 2.5% NaOCl: NCF = 385.5 (SD = 76.03), Fragment length = 2.4 mm (SD = 0.5) 5 min 2.5% NaOCl + sterilization: NCF = 359 (SD = 73.25), Fragment length = 2.5 mm (SD = 0.6) 5× (5 min 2.5% NaOCl + sterilization): NCF = 320 (SD = 61.55), Fragment length = 2.6 mm (SD = 0.2) FlexMaster (n = 40): Control: NCF = 379 (SD = 70.54), Fragment length = 3 mm (SD = 0.4) 5 min 2.5% NaOCl: NCF = 378 (SD = 53.39), Fragment length = 2.5 mm (SD = 0.4) 5 min 2.5% NaOCl + sterilization: NCF = 374 (SD = 70.82), Fragment length = 2.7 mm (SD = 0.3) 5× (5 min 2.5% NaOCl + sterilization): NCF = 373.5 (SD = 67.37), Fragment length = 2.5 mm (SD = 0.4) Twisted File (n = 40): Control: NCF = 551.2 (SD = 118.78), Fragment length = 2.4 mm (SD = 0.4) 5 min 2.5% NaOCl: NCF = 526.5 (SD = 105.8), Fragment length = 2.4 mm (SD = 0.2) 5 min 2.5% NaOCl + sterilization: NCF = 439 (SD = 148.16), Fragment length = 2.6 mm (SD = 0.4) 5× (5 min 2.5% NaOCl + sterilization): NCF = 415.7 (SD = 126.21), Fragment length = 2.5 mm (SD = 0.2) Mtwo (n = 40): Control: NCF = 599.5 (SD = 79.46), Fragment length = 2.8 mm (SD = 0.2) 5 min 2.5% NaOCl: NCF = 560 (SD = 56.81), Fragment length = 2.7 mm (SD = 0.2) 5 min 2.5% NaOCl + sterilization: NCF = 553 (SD = 87.94), Fragment length = 2.6 mm (SD = 0.2) 5× (5 min 2.5% NaOCl + sterilization): NCF = 514.5 (SD = 73.76), Fragment length = 2.6 mm (SD = 0.2)	None
Pedulla [38]	90 files 45 per brand 15 per group Reciproc R25 and WaveOne Primary	Group 1 (control) new instruments Group 2 immersion in sodium hypo 5%, 37 °C, 1 min Group 3 immersion in sodium hypo 5%, 37 °C, 5 min	The artifi- cial canal was manufactured by reproducing an instrument’s size and taper. It provided the instrument with a suitable simulated root canal with a 60° angle of curvature and 5 mm radius of curvature measured according to the method of Schneider The center of the curvature was 6 mm from the tip of the instrument,	RECIPROC ALL and WAVEONE ALL	dynamic immersion, with a 6:1 reduction endodontic motor After rinsing and drying all three groups of each brand were tested in fatigue test	Time to fracture (in seconds) RECIPROC, Group 1: Mean TtF was 119.7 s. Median = 121 s. Standard deviation = 17.72. Group 2: Mean TtF = 107.0 s. Median = 106 s. Standard deviation = 18.94. Group 3: Mean TtF = 108.8 s. Median = 111 s. Standard deviation = 17.27. WAVEONE, Group 1: Mean TtF = 74.8 s. Median = 74 s. Standard deviation = 14.59. Group 2: Mean TtF = 70.5 s. Median = 68 s. Standard deviation = 13.60. Group 3: Mean TtF = 71.0 s. Median = 73 s. Standard deviation = 14.92.	None
Cai [39]	25.06 HyFlex and M3 AFM testing: 4 files 2 per brand Fatigue test 72 files 36 files per brand 12 er subgroup	AFM test dynamic immersion in 5.25% sodium hypochlorite or 17% (EDTA) solution for 10 min Fatigue test 5.25% NaOCl Group 1—no immersion, Group 2—dynamic immersion in 5.25% sodium hypochlorite for 10 min Group 3—17% (EDTA) solution for 10 min	curvature of 45° with a 3 mm radius	400 or 500 rpm	Dynamic immersion in 5.25% NaOCl for 10 min and Groups 3 were dynamic Immersion in 17% EDTA for 10 min. Fatigue test synthetic oil was sprayed into the simulated canal. Test performed at room temperature.	HyFlex Mean Roughness Ra (nm) New, Mean (Ra): 1441, SD: 373 5.25% NaOCl, Mean (Ra): 1412, SD: 324 17% EDTA, Mean (Ra): 1566, SD: 405 M3 New, Mean (Ra): 1203, SD: 155 5.25% NaOCl, Mean (Ra): 1235, SD: 295 17% EDTA, Mean (Ra): 1269, SD: 258	AFM atomic force microscopy Significant differences in surface roughness after immersion were observed between the two files.
Cheung [15]	ProFile—NiTi engine-file (size 25, 0.04 and 0.06 taper)	Sodium hypochlorite: 1.2% aqueous solution, 23 °C ± 2 °C Deionized water: 23 °C ± 2 °C Air: relative humidity 65%, 23 °C ± 2 °C Silicone oil: “for oil bath”, 23 °C ± 2 °C Exposure: Only the curved portion of the instrument was immersed during testing to avoid galvanic action	Static model using 3 rigid stainless steel pins. Various curvatures tested. Variable radius of curvature (Rc) determined.	250 rpm, continuous rotational motion	Custom-made rotational-bending fatigue testing machine. The test employed a strain-life approach with continuous rotation at 250 rpm until fracture, with electronic monitoring via optical counter and break-detection circuit. Pecking motion not tested.	air (106 total, 95 failed in LCF), water (106 total, 87 failed in LCF), hypochlorite (39 total, 30 failed in LCF), and silicone oil (40 total, 36 failed in LCF). In the low-cycle fatigue (LCF) region, fatigue life ranged from approximately 100–10,000 cycles before transitioning to high-cycle fatigue at ~1.2% strain amplitude around 2000–10,000 cycles. The regression line slopes for LCF lives were: air (−1.603 ± 0.104), water (−1.190 ± 0.127), hypochlorite (−1.196 ± 0.137), and silicone oil (−1.555 ± 0.128).	Crack origins, fatigue striations, and dimple rupture regions on all specimens. Corrosion assessment: Only 1 specimen in hypochlorite group showed corrosion pits; no corrosion pits found in water, air, or silicone oil groups Crack initiation sites: almost all specimens showed crack initiation at cutting edge or “radial land” region.
Elnaghy [40]	WaveOne Gold size 25, 0.07 taper (Gold wire thermal treatment alloy) Reciproc size 25, 0.08 taper (M-Wire NiTi)	Sodium hypochlorite: 5% NaOCl, 37 °C ± 1 °C Saline: 37 °C ± 1 °C Air: control group at room temperature 20 °C ± 1 °C Exposure: Instruments were immersed in test solutions during reciprocation until fracture	Static model using custom-made device with 3 stainless steel pins Curvature angle: 60° Radius: 5 mm radius of curvature Position: Center of curvature was 5 mm from instrument tip Working length: 19 mm for each instrument	Reciprocating motion, WaveOne ALL for WaveOne Gold and Reciproc ALL for Reciproc	Custom-made device with three stainless steel pins (6 mm diameter, 4 cm long) attached to a Teflon base with 0.5 mm wide V-shaped notches to preserve instrument location during rotation, placed inside a glass container and stabilized with clamps. Instruments were reciprocated in test solutions until fracture, with time to failure recorded in seconds and converted to cycles by multiplying by rotational speed.	WaveOne Gold achieved 1505.9 ± 163.75 cycles in air, 1066.0 ± 113.93 cycles in saline, and 990.83 ± 106.93 cycles in NaOCl. Reciproc achieved 1251.03 ± 103.29 cycles in air, 912.83 ± 100.69 cycles in saline, and 880.27 ± 50.79 cycles in NaOCl. WaveOne Gold had 1027 predicted cycles for 99% survival in air, while Reciproc in NaOCl had the lowest predicted cycles (613 cycles). Fragment lengths were consistent around 5 mm from tip across all groups with no significant differences.	Crack origins, fatigue zones, and overload fast fracture zones in all specimens. Crack initiation occurred at the cutting edge in all instruments. Multiple fatigue-crack initiation locations were found in Reciproc fractured fragments, WaveOne Gold had improved resistance to fatigue-crack initiation. No pitting corrosion was observed.
Keles [41]	Reciproc 25- traditional NiTi Reciproc Blue R25-heat-treated NiTi WaveOne Primary-traditional NiTi WaveOne Gold Primary—heat-treated NiTi One Shape (25.06)—traditional NiTi	Sodium hypochlorite (5.25% NaOCl) Distilled water (DW) Temperature: 37 °C ± 1 °C and 60 °C ± 1 °C Exposure: 5 min immersion period	Dynamic model (with axial motion) Angle of curvature: 60° Radius: 5 mm Material: Stainless steel artificial canal	Reciproc and Reciproc Blue: Preset “Reciproc” program on X-Smart Plus motor WaveOne and WaveOne Gold: Preset “WaveOne” program on X-Smart Plus motor One Shape: 350 rpm and 2.5 N/cm torque Axial motion: 3 mm amplitude approximately every 2 s (pecking motion)	Dynamic cyclic fatigue test using a specialized cyclic fatigue testing device. Instruments rotated freely until fracture occurred.	TtF (in sec) WaveOne: -Control (no immersion): 157 ± 32 s -Distilled water at 37 °C: 161 ± 6 s -Distilled water at 60 °C: 198 ± 24 s -NaOCl at 37 °C: 164 ± 39 s -NaOCl at 60 °C: 21 ± 10 s WaveOne Gold: -Control (no immersion): 256 ± 63 s -Distilled water at 37 °C: 266 ± 33 s -Distilled water at 60 °C: 208 ± 47 s -NaOCl at 37 °C: 222 ± 42 s -NaOCl at 60 °C: 139 ± 81 s Reciproc: -Control (no immersion): 241 ± 67 s -Distilled water at 37 °C: 277 ± 44 s -Distilled water at 60°C: 312 ± 65 s -NaOCl at 37 °C: 91 ± 27 s -NaOCl at 60 °C: 50 ± 20 s Reciproc Blue: -Control (no immersion): 259 ± 99 s -Distilled water at 37 °C: 314 ± 98 s -Distilled water at 60 °C: 536 ± 106 s -NaOCl at 37 °C: 316 ± 65 s -NaOCl at 60 °C: 301 ± 78 s OneShape: -Control (no immersion): 153 ± 32 s -Distilled water at 37 °C: 142 ± 14 s -Distilled water at 60 °C: 163 ± 31 s -NaOCl at 37 °C: 112 ± 11 s -NaOCl at 60 °C: 85 s	Typical cyclic fatigue failure patterns in all tested instruments. Clear corrosion areas were visible, especially on OneShape instruments after 5 min of immersion in NaOCl at 60 °C. All fractures were consistent with cyclic fatigue failure mechanisms across all test conditions.
Alfawaz [42]	F2 size (#25) with 8% taper, 25 mm ProTaper Gold (PTG)—heat-treated NiTi EdgeTaper Platinum (ETP)—heat-treated NiTi	17% EDTA, 5.25% NaOCl, and Distilled water (control) Temperature: 37 °C ± 1 °C Exposure: Continuous immersion during testing (instruments rotated within the solution-filled canal)	Static artificial canals milled in stainless steel blocks. Angle of curvature: 60° Radius: 5 mm Curvature center: 5 mm from instrument tip	300 rpm, continuous rotation until fracture. Insertion depth: 19 mm from instrument tip.	Dynamic cyclic fatigue test using artificial canals. Instruments rotated continuously until fracture occurred. Glass bar used to cover canal and prevent slippage. Fracture detected audibly and visually with video recording assistance.	NCF: ProTaper Gold: EDTA: 979.0 ± 184.9 cycles NaOCl: 659.4 ± 111.8 cycles Distilled water: 939.4 ± 176.9 cycles EdgeTaper Platinum: EDTA: 1862.3 ± 365.7 cycles NaOCl: 1422.8 ± 246.6 cycles Distilled water: 1664.5 ± 285.9 cycles	Typical cyclic fatigue failure patterns in all tested instruments. Presence of fatigue striations and crack initiation areas. Fast fracture zone with dimples visible. No indication of corrosion observed in any of the tested solutions (17% EDTA, 5.25% NaOCl, or water).
Tyagi [43]	25 mm length GenEndo—heat-treated NiTi T-wire ProTaper Gold (Dentsply Sirona, New Delhi, India)—heat-treated NiTi Hero Gold—NiTi EdgeFile X3—heat-treated NiTi with annealing treatment	Distilled water (control), 2.5% NaOCl, and 5.25% NaOCl Temperature: 25 °C, 37 °C, and 60 °C Exposure: Continuous immersion during testing (instruments rotated within the solution-filled canal)	Static artificial canals prepared in stainless steel blocks. Angle of curvature: 60° Radius: 5 mm Curvature center: 5 mm from instrument tip	300 rpm, continuous rotation. Insertion depth: 19 mm from instrument tip	Dynamic cyclic fatigue test using artificial canals. Fixed and reproducible positioning using a mounted handpiece. Glass cover used to prevent instrument slippage and visualize fracture timing.	NCF GenEndo: -Distilled water: 1245.2 ± 382.3 (25 °C), 951.8 ± 289.1 (37 °C), 721.6 ± 112.6 (60 °C) -2.5% NaOCl: 1123.4 ± 265.7 (25 °C), 874.6 ± 227.9 (37 °C), 689.8 ± 117.8 (60 °C) -5.25% NaOCl: 1045.6 ± 287.1 (25 °C), 766.2 ± 192.8 (37 °C), 542.7 ± 91.9 (60 °C) ProTaper Gold: -Distilled water: 1139.1 ± 277.3 (25 °C), 912.9 ± 276.0 (37 °C), 367.7 ± 116.9 (60 °C) -2.5% NaOCl: 721.3 ± 270.9 (25 °C), 602.3 ± 225.6 (37 °C), 305.7 ± 116.7 (60 °C) -5.25% NaOCl: 817.7 ± 296.1 (25 °C), 551.1 ± 174.4 (37 °C), 285.4 ± 90.8 (60 °C) Hero Gold: -Distilled water: 1106.2 ± 266.3 (25 °C), 862.9 ± 276.0 (37 °C), 329.3 ± 121.7 (60 °C) -2.5% NaOCl: 693.2 ± 260.6 (25 °C), 552.3 ± 207.9 (37 °C), 275.9 ± 116.9 (60 °C) -5.25% NaOCl: 786.5 ± 281.1 (25 °C), 518.1 ± 172.3 (37 °C), 254.6 ± 90.6 (60 °C) EdgeFile X3: -Distilled water: 1058.6 ± 277.1 (25 °C), 822.7 ± 243.1 (37 °C), 278.7 ± 115.9 (60 °C) -2.5% NaOCl: 641.2 ± 169.8 (25 °C), 513.7 ± 216.8 (37 °C), 214.8 ± 147.6 (60 °C) -5.25% NaOCl: 744.4 ± 298.0 (25 °C), 476.6 ± 141.4 (37 °C), 194.8 ± 96.7 (60 °C)	Typical cyclic fatigue failure patterns in all tested instruments. Crack initiation areas identified. Consistent fracture morphology across all testing conditions. Evidence of micropitting due to NaOCl corrosive effects, particularly with higher concentrations and temperatures.
Erik [44]	-Reciproc blue -Wave One Gold -HyFlex EDM	Temperature of solutions—37 ± 1 °C. Irrigants: -distilled water (control) -6% NaOCl -17% EDTA -+8% HEBP; -3% NaOCl + 9% HEBP	The size of the artificial canals: -25/0.08(RPC Blue and HEDM),—25/0.07 (WO Gold) Curvature: 60*°*; 5 mm radius.	Reciproc GOLD endodontic motor, -“WaveOne ALL” program for WOG (reciprocal movement, 350 rpm) Reciproc ALL” program for “RPC Blue”(reciprocal movement, 300 rpm) −500 rpm and 2.5 N·cm torque values for HEDM	The files were rotated in the custom-made artificial canals according to manufacturer’s recommendations, until fracture occurred.	Number of cycles to failure (NCF) and fracture length (FL) of instruments 1. Reciproc Blue: (a) Distilled water NCF: 1543.9 ± 185.1 ax FL: 5.2 ± 0.5 (b) 6% NaOCl NCF: 1449.1 ± 202.8 ax FL5.2 ± 0.5 (c) 17% EDTA NCF: 1468.5 ± 190.8 ax FL: 5.1 ± 0.4 (d) 18% HEBP NCF: 1409.2 ± 225.4 ax FL:4.9 ± 0.4 (e) 18% HEBP + 6% NaOCl NCF: 1289.9 ± 141.7 ay FL: 5.2 ± 0.4 *p* value < 0.05 (for NCF) > 0.05 (for FL) 2. WaveOneGold (a) Distilled water NCF: 1489.8 ± 208.4 ax FL: 5.2 ± 0.3 (b) 6% NaOCl NCF: 1441.5 ± 172.9 ax FL: 5.1 ± 0.4 (c) 17% EDTA NCF: 1444.4 ± 173.2 ax FL: 4.9 ± 0.5 (d) 18% HEBP NCF: 1367.1 ± 177.7 ax FL: 5.3 ± 0.5 (e) 18% HEBP + 6% NaOCl NCF: 1015.1 ± 152.2 ay FL: 5.2 ± 0.4 *p* value < 0.05 (for NCF) > 0.05 (for FL) 3. HyFlex EDM (a) Distilled water NCF: 1712.3 ± 188.3 bx FL: 5.3 ± 0.4 (b) 6% NaOCl NCF: 1662.1 ± 249.3 bx FL: 5.1 ± 0.4 (c) 17% EDTA NCF: 1672.7 ± 217.3 bx FL: 5.1 ± 0.6 (d) 18% HEBP NCF: 1655.2 ± 198.6 bxFL: 5.2 ± 0.5 (e) 18% HEBP + 6% NaOCl NCF: 1412.3 ± 169.4 by FL: 5.2 ± 0.4 *p* value < 0.05 (for NCF) > 0.05 (for FL)	The SEM analysis showed typical features of the fractured cross-sectionssurfaces, (fatigue zones, crack origins, overload zones).
Pedulla [45]	150 new Twisted Files (SybronEndo, Orange, CA, USA), Revo S SU files (Micro Mega, Besancon, France), Mtwo files (Sweden & Martina, Padova, Italy), Size 25.06	5% NaOCl (Niclor, OGNA Laboratory, Milan, Italy), 37 °C	60 degree angle of curvature and a 5 mm radius of curvature. The center of the curvature was 6 mm from the tip of the instrument, and the curved segment of the canal was approximately 6 mm in length.	Constant speed of 300 rpm. 6:1 reduction handpiece (Sirona Dental Systems GmbH, Ensheim, Germany) powered by a torque-controlled electric motor (VDW Silver, VDW GmbH–Dentsply International Inc, Munich, Germany). Torque was set at 2 N·cm.	High-flow synthetic oil (Super Oil; Singer Co Ltd., Elizabethport, NJ, USA) was applied. TtF was recorded with a chronometer to an accuracy of 0.1 s. NCF were calculated to the nearest full number ultiplying the seconds by 5 (number of cycles for second using 300 rpm).	Twisted File Group 1 (n = 10): (a) Mean (seconds)-742.5 (b) Median-750 (c) Standard deviation-87.38 (d) Standard error of mean-27.63 (e) Min-555 (f) Max-865 Group 2 (n = 10): (a) Mean (seconds)-729.5 (b) Median-750 (c) Standard deviation-121.7 (d) Standard (error of mean-38.5 (e) Min-575 (f) Max-915 Group 3 (n = 10): (a) Mean (seconds)-703 (b) Median-735 (c) Standard deviation-92.26 (d) Standard error of mean-29.18 (e) Min-520 (f) Max-795 Group 4 (n = 10): (a) Mean (seconds)-802.5 (b) Median-807.5 (c) Standard deviation-84.73 (d) Standard error of mean-26.79 (e) Min-710 (f) Max-975 Group 5 (n = 10): (a) Mean (seconds)-755 (b) Median-767.5 (c) Standard deviation-138.9 (d) Standard error of mean-43.92 (e) Min-450 (f) Max-915 Revo S SU Group 1 (n = 10): (a) Mean (seconds)-395.5 (b) Median-375 (c) Standard deviation-72.17 (d) Standard error of mean-22.82 (e) Min-315 (f) Max-565 Group 2 (n = 10): (a) Mean (seconds)-417.5 (b) Median-452.5 (c) Standard deviation-124.3 (d) Standard error of mean-39.32 (e) Min-155 (f) Max-540 Group 3 (n = 10): (a) Mean (seconds)-392 (b) Median-390 (c) Standard deviation-92.14 (d) Standard error of mean-29.14 (e) Min-275 (f) Max-545 Group 4 (n = 10): (a) Mean (seconds)-390 (b) Median- 367.5 (c) Standard deviation-92,33 (d) Standard error of mean-29.2 (e) Min-260 (f) Max-530 Group 5 (n = 10): (a) Mean (seconds)-382 (b) Median- 390 (c) Standard deviation-35.76 (d) Standard error of mean-11.31 (e) Min-305 (f) Max-420 Mtwo Group 1 (n = 10): (a) Mean (seconds)-560 (b) Median-540 (c) Standard deviation-107.5 (d) Standard error of mean-33.99 (e) Min-450 (f) Max-730 Group 2 (n = 10): (a) Mean (seconds)-533 (b) Median-480 (c) Standard deviation-122 (d) Standard error of mean-38.59 (e) Min-380 (f) Max-700 Group 3 (n = 10): (a) Mean (seconds)-563 (b) Median-515.5 (c) Standard deviation-145.5 (d) Standard error of mean-46.01 (e) Min-435 (f) Max-905 Group 4 (n = 10): (a) Mean (seconds)-579.5 (b) Median-552.5 (c) Standard deviation-113 (d) Standard error of mean-35.73 (e) Min-450 (f) Max-800 Group 5 (n = 10): (a) Mean (seconds)-571.5 (b) Median-572.5 (c) Standard deviation-145.8 (d) Standard error of mean-46.12 (e) Min-415 (f) Max-840	There was no SEM analysis
Dosanjh [46]	30 files per subgroup EdgeEndo, Vortex Blue; ESX size 25 with a 0.04	4 subgroups 1—water at 3 °C, 2—water at 22 °C, 3—water at 37 °C, 4—water at 60 °C.	canal curvature of 60 degree and a 5 mm radius curvature.	Rotation at 500 rpm	The block was submerged in a water bath. Immersion during fatigue test.	NCF: -Edge Endo 3—6185 22—7243 37—1675 60—901 -Vortex 3—4842 22—2062 37—1233 60—651 -ESX 2—932 22—466 37—271 60—218	No data

AFM—Atomic Force Microscopy, °C—degree Celsius, EDTA—Ethylenediaminetetraacetic Acid, h—hour(s), min—minute(s), mm—millimetre(s), NaOCl—Sodium Hypochlorite, NCF—Number of Cycles to Fracture, nm—nanometre(s), N·cm—Newton centimetre, Ra—Roughness average, rpm—revolutions per minute, s—second(s), SD—Standard Deviation, SEM—Scanning Electron Microscopy.

### 3.4. Quality Assesment

For all of the 9 questions, 3 papers received a positive answer to 9 of them [22,23,25], 23 papers received a positive answer to 8 of them [15,24,27,29,30,31,32,33,34,35,36,37,38,39,40,41,42,43,44,45,46,47] and 1 paper received a positive answer to 7 of them [26] (see Table 3).

## 4. Discussion

The aim of this systematic review was to evaluate the effect of various endodontic irrigants on the cyclic fatigue resistance (CFR) of nickel–titanium (NiTi) rotary instruments. The included studies examined the influence of different irrigants—primarily sodium hypochlorite (NaOCl)—on the fatigue performance of NiTi files under varying experimental conditions. In the majority of studies [22,23,24,25,27,28,29,30,32,35,36,39,40,43,44,45,47], the use of NaOCl at concentrations of 5.25%, particularly when combined with elevated temperatures, was associated with a significant reduction in CFR. Berutti [24], Alfawaz [27], and Tyagi [43] specifically demonstrated that higher concentrations and temperatures of NaOCl accelerated instrument fracture. Scanning electron microscopy (SEM) analyses conducted in several studies [15,22,23,24,27,30,43,44,47] revealed characteristic features of cyclic fatigue failure, including crack initiation sites, fatigue striations, and ductile fracture zones. In many cases, surface corrosion was evident after exposure to NaOCl, whereas other irrigants produced no noticeable surface alterations. In summary, the findings of this review indicate that NaOCl, especially at high concentrations and elevated temperatures, has a detrimental effect on the cyclic fatigue resistance of NiTi rotary instruments. In contrast, alternative irrigants such as EDTA or saline appear to exert a more neutral influence, particularly on heat-treated NiTi alloys.

The fracture resistance of endodontic instruments is strongly influenced by the metallurgical properties of the alloy and the specific thermal treatments applied during the manufacturing process [48]. Conventional nickel–titanium (NiTi) alloys, which predominantly exist in the austenitic phase at body temperature, tend to exhibit lower flexibility and greater susceptibility to cyclic fatigue, particularly in curved canals [48,49,50]. Multiple studies supporting the present findings have demonstrated that modern heat-treated NiTi files—such as those made from M-Wire, CM-Wire, Gold, and Blue alloys—offer enhanced flexibility and improved resistance to cyclic fatigue [3,49,50,51]. These properties enable the instruments to better adapt to complex canal anatomies, thereby reducing the risk of instrument separation during root canal preparation.

The choice of irrigant type plays a critical role in the durability of NiTi rotary instruments. Sodium hypochlorite, while widely used for its potent antimicrobial activity and tissue-dissolving capabilities, has been shown to compromise the structural integrity of NiTi files by promoting surface corrosion and initiating microstructural defects such as microcracks [49,50]. In a meta-analysis, dos Reis-Prado et al. reported that sodium hypochlorite concentrations ≥5% and temperatures ≥37 °C significantly reduced the fatigue life of NiTi instruments, particularly those made from conventional alloys, resulting in a marked decrease in the number of cycles to fracture (NCF) [50]. These findings are consistent with the data presented in the current review and underscore the need to consider not only the antimicrobial efficacy of irrigants but also their impact on instrument longevity during endodontic procedures.

The type of kinematic motion—rotary versus reciprocating—plays a critical role in the stress distribution and fatigue resistance of NiTi endodontic instruments, particularly in curved canals. Systematic reviews have demonstrated that reciprocating motion significantly enhances cyclic fatigue resistance compared with continuous rotation, as measured by the number of cycles to fracture (NCF) [52]. To emphasize this difference, it is valuable to present available data. A study by Keles et al. [41] demonstrated that the time to fracture (TtF, in seconds) was more than four times higher in 60 °C heated NaOCl for Reciproc Blue (301 s) compared with OneShape (85 s). Similarly, Kermeoglu et al. [33] reported an average TtF of 20.89 s for the ProTaper Universal file across all tests, compared with 112.75 s for Reciproc M-Wire. Independent of canal curvature angle or radius, reciprocating motion appears to reduce torsional stress and delay the onset of fatigue failure more effectively than rotary motion [5,53]. This advantage is particularly relevant in anatomically challenging cases, such as sharply curved or S-shaped canals, where repetitive flexion near the apical curvature accelerates fatigue-related damage during continuous rotation.

Several limitations of this systematic review should be taken into account when interpreting the findings. Firstly, all included studies were conducted under in vitro conditions, which do not fully replicate the complexity of the clinical environment. Factors such as intracanal temperature fluctuations, the presence of organic tissues, and natural root canal curvature are difficult to simulate accurately, potentially limiting the clinical applicability of the results. Secondly, this review included only studies published in English, which may have introduced language bias and led to the exclusion of relevant research reported in other languages. This restriction, however, was necessary to ensure accurate interpretation and critical appraisal of methodological details. Additionally, while scanning electron microscopy (SEM) was frequently employed to evaluate surface degradation and fracture characteristics, inconsistencies in imaging protocols and evaluation criteria across studies may have contributed to variability in the results. Moreover, the methodological quality of the included studies, as assessed using the JBI checklist for quasi-experimental studies (9 criteria in total), showed some variation: three studies fulfilled all 9/9 criteria [22,23,25], twenty-three studies fulfilled 8/9 criteria [15,24,27,28,29,30,31,32,33,34,35,36,37,38,39,40,41,42,43,44,45,46,47], and one study fulfilled 7/9 criteria [26]. This variation highlights another constraint in the quality of the studies that may have impacted the reliability and consistency of the results. To enhance the quality and relevance of future research, it is recommended that investigators adopt standardized in vitro protocols that more closely mimic clinical conditions. This includes the use of consistent cyclic fatigue testing parameters, uniform SEM evaluation methods, and, when feasible, natural human teeth to better reflect clinical performance. Expanding the language scope in systematic literature searches may also increase the comprehensiveness and generalizability of future reviews.

Given the available data and the absence of a clear and reproducible testing protocol, the need for greater standardization can be assumed. From a clinical perspective, two key groups of factors require particular consideration: canal curvature and the chemical environment. To closely simulate clinical conditions, all irrigants—especially sodium hypochlorite—should be maintained at a constant temperature of 37 °C. Furthermore, a 60° curvature with a 5 mm radius represents the most commonly employed configuration of artificial canals across studies and may therefore serve as a suitable standard for future investigations.

## 5. Conclusions

Within the limitations of the reviewed data, it was confirmed that sodium hypochlorite—especially at concentrations of 5% or higher and at elevated temperatures, including body temperature—was the most corrosive irrigant, negatively affecting the cyclic fatigue resistance of nickel–titanium (NiTi) rotary instruments. Scanning electron microscopy (SEM) revealed surface corrosion and fatigue-related structural changes in most studies following exposure to NaOCl. Heat-treated NiTi instruments showed significantly greater resistance to cyclic fatigue compared to conventional alloys in the majority of studies. Short-term immersion in NaOCl (1–5 min) did not result in statistically significant reductions in fatigue resistance; however, when combined with repeated autoclave sterilization, a cumulative weakening effect on the alloy was observed. These findings highlighted the importance of considering both irrigant selection and clinical protocols to preserve the mechanical integrity and performance of NiTi instruments during root canal treatment. A thorough analysis of the available data leads to a practical conclusion: files subjected to multiple sterilization cycles and/or exposure to sodium hypochlorite at higher concentrations may exhibit surface alterations and increased fragility. Consequently, the reuse of such instruments should be approached with caution, particularly in anatomically challenging cases. From a clinical perspective, we recommend that clinicians (i) limit the number of reuse cycles for NiTi instruments, (ii) avoid reusing files previously exposed to high NaOCl concentrations in complex canals, and (iii) routinely inspect instruments under magnification before reuse. Adoption of these measures may reduce the risk of unexpected fracture and improve patient safety.

## Figures and Tables

**Table 1:** General characteristic on included studies.

Study	Aim of the Study	Materials and Methods	Results	Conclusions
de Castro Martins [22]	How 5.25% NaOCl impacts the surface conditions and fatigue resistance of Profile Ni-Ti instruments.	80 instruments were split into one control group (CG) and three experimental groups (EG1,2,3). In the EG1 instruments were soaked in NaOCl for 24 h. In the EG2 and 3 instruments were used to prepare curved root canals—one with water and the other one with NaOCl during the procedure.	SEM analysis revealed surface wear (scratches, metal loss) without corrosion in used instruments. NaOCl-treated instruments (EG3) showed significantly faster performance than untreated ones (EG2). Both groups developed microcracks 2.2–3.8 mm from the tip. Canal-used instruments (EG2,3) demonstrated significantly reduced fatigue resistance versus unused controls (CG, EG1). Fractures typically occurred ~4 mm from the tip due to fatigue failure, confirmed by SEM.	Soaking in 5.25% NaOCl for 24 h did not change the instrument’s surface or fatigue resistance. The tools that were used in the simulated root canal procedures had reduced fatigue resistance.
Cheung [28]	Evaluation the low-cycle fatigue response of NiTi rotary instruments working with NaOCl solution.	179 instruments from 4 different brands were tested by rotating them at 250 rpm in a 1.2% NaOCl solution until they fractured. Surface strain was calculated from instrument curvature and fracture diameter, then compared to the number of rotations until failure.	All groups showed a linear strain-life relationship. The fatigue-ductility was similar across brands, but the number of crack initiation points differed. A inverse linear correlation was found between crack growth and strain amplitude.	NiTi rotary instruments in NaOCl show both low- and high-cycle fatigue patterns. The instrument’s cross-sectional shape does not influence LCF behavior. A certain level of crack growth leads to sudden fracture depending on strain amplitude.
Ormiga Galvão Barbosa [23]	Effect of NaOCl on the Mechanical Behavior of K3 NiTi Rotary Files	Both new and NaOCl –treated files were evaluated for their resistance to flexural fatigue and torsional fracture. A *t*-test was applied to compare the groups in terms of the number of cycles, rotation angle, and peak torque leading to fracture.	There was no statistically significant variation among the groups. SEM analysis revealed no signs of localized corrosion on the files that had been exposed to NaOCl.	The findings indicate that contact with NaOCl does not affect the fracture resistance of K3 rotary files.
Berutti [24]	To assess how immersion in NaOCl affects the cyclic fatigue resistance and corrosion behavior of ProTaper NiTi rotary files.	120 ProTaper NiTi F2 files were randomly allocated into three groups (n = 40 each). Group 1 served as control. Group 2 had 20 mm of each file (excluding shaft) immersed in 5% NaOCl at 50 °C for 5 min. Group 3 had complete file submersion under identical conditions. Prematurely fractured Group 3 instruments underwent field emission SEM for micromorphological and microchemical analysis.	The files from group 3 demonstrated notably reduced fracture resistance due to cyclic fatigue compared to those in groups 1 and 2 (*p* < 0.001). In certain cases, instruments in group 3 fractured within just a few seconds of fatigue testing. SEM analysis showed clear indications of corrosion on the broken instruments.	Group 3 instruments demonstrated significantly reduced cyclic fatigue resistance compared to Groups 1 and 2. Early fractures may result from galvanic corrosion caused by dissimilar metals, where one metal acts as the cathode in NaOCl solution while the NiTi alloy serves as the anode, increasing corrosion susceptibility.
Javadi [29]	To investigate the effect of immersion in 5% NaOCl and Deconex on the cyclic fatigue resistance of two NiTi rotary instruments.	90 rotary files (M3 Pro Gold 25.06 and SP1 F2) were used, with 45 instruments per type randomly divided into three groups (n = 15). Groups underwent 5 min room temperature exposure to: no immersion (control), 5% NaOCl, or Deconex. Cyclic fatigue resistance was assessed using a custom testing device.	The two-way ANOVA showed a significant difference in average cyclic fatigue resistance between M3 and SP1 files. The M3 files treated with NaOCl had the lowest resistance, while SP1 files exposed to Deconex showed the highest. Both the type of disinfectant (*p* < 0.001) and the file type (*p* < 0.001) had a statistically significant impact on fatigue resistance.	Immersing NiTi rotary instruments in disinfectant solutions can influence their resistance to cyclic fatigue, and the degree of this effect depends on the file type and the type of disinfectant applied.
Palma [30]	To evaluate and compare the cyclic fatigue resistance of three different rotary file systems in a dynamic model after immersion in 3% NaOCl.	90 from 3 NiTi systems (ProTaper Next, Hyflex CM, Hyflex EDM) were randomly assigned to nine groups based on file type and 3% NaOCl immersion time (0, 1, or 5 min). Cyclic fatigue testing was performed in a simulated root canal (45° curvature, 5 mm radius) until fracture. Instrument reliability was assessed using Weibull statistical method.	PTN instruments averaged 1200 ± 178 cycles to fracture, CM reached 1949 ± 362, and EDM achieved 5573 ± 853 cycles—a statistically significant difference (*p* < 0.01). Immersion in 3% NaOCl led to a significant decrease in the average number of cycles to fracture (*p* = 0.01).	EDM instruments showed the highest fatigue resistance, followed by CM. PTN had the shortest lifespan. Sodium hypochlorite reduced fatigue resistance in all files, especially in CM-wire instruments.
Priya [31]	Evaluation of the impact of the autoclave sterilisationand sodium hypochlorite immersion on the cyclic fatigue resistance of four different NiTi rotary instruments.	A total of 320 new instruments were divided into four subgroups: Control (no treatment); Immersion in 5.25% NaOCl for 3 min; Three autoclave sterilization cycles; Combined immersion in NaOCl + three sterilizations. Each instrument was testednfor cyclic fatigue in a simulated curved canal setup, and the number of cycles to failure (NCF) was recorded.	EdgeFile X3 consistently showed the highest fatigue resistance across all treatment types. It was followed by Hyflex EDM, Hyflex CM, and TruNatomy, which had the lowest resistance. Neither NaOCl immersion alone nor sterilization alone significantly reduced cyclic fatigue resistance. The combination of both treatments actually increased NCF, suggesting a possible strengthening effect under those conditions.	All four file systems showed some resilience to treatment-related stress. EdgeFile X3 was the most durable, while TruNatomy was the most susceptible to fatigue failure. Immersion in NaOCl and multiple sterilization cycles did not negatively affect, and in some cases improved, the fatigue resistance of these heat-treated instruments.
Mousavi [25]	To investigate whether the temperature of sodium hypochlorite (NaOCl)—a common root canal irrigation solution—affects the cyclic fatigue resistance of ProTaper Gold (PTG) rotary endodontic files.	45 PTG S1 rotary files were tested in an artificial canal (60° curvature, 5 mm radius) divided into three groups by NaOCl temperature: 22 °C (room temperature), 4 °C (cold), and 37 °C (body temperature). Files rotated at 300 rpm until fracture. Number of cycles to fracture (NCF) and fragment length were recorded.	There was no statistically significant difference in cyclic fatigue resistance or fragment length among the three groups. Thus, temperature variation between 4 °C and 37 °C did not significantly affect the performance of PTG files.	The cyclic fatigue resistance of heat-treated PTG rotary files is not significantly influenced by the temperature of the NaOCl solution within the tested range. Further clinical studies are recommended.
Tanomaru [47]	To evaluate the cyclic fatigue resistance of the XP-Endo Finisher (XPF) instrument in a dynamic model, using two different irrigation solutions: 2% chlorhexidine gel (CHX) and 6% sodium hypochlorite (NaOCl).	30 new XPF files (25 mm long, 0.25 mm tip diameter) were divided into three groups (n = 10 each) based on irrigation solution: Group 1: 2% chlorhexidine gel (CHX), Group 2: 6% NaOCl, Group 3 (control): lubricating oil (WD-40). Stainless-steel artificial canal simulated severe apical curvature (3 mm, 90°).	CHX group demonstrated significantly higher cyclic fatigue resistance (4.99 min, 4558 cycles to fracture) compared to NaOCl group (0.92 min, 1014 cycles) and oil group (0.67 min, 832 cycles). CHX significantly outperformed both solutions (*p* < 0.001), with no significant difference between NaOCl and oil.	XP-Endo Finisher showed longest fatigue life with 2% CHX due to its lubricating and non-corrosive properties, unlike NaOCl which may corrode NiTi alloy and reduce fatigue resistance. While CHX improves resistance, it cannot dissolve organic tissue and thus cannot replace NaOCl. Further studies should explore alternative irrigants.
Abuhulaibah [32]	To evaluate cyclic fatigue (CF) resistance of two NiTi endodontic files (One Curve, ProTaper Gold) after immersion in 2.5% NaOCl at 37 °C for different time periods	45 files of each type (OC, PTG) were divided into three groups: no immersion (control), 1 min NaOCl immersion, and 5 min NaOCl immersion. CF testing was performed in a simulated curved canal at 37 °C water bath. Number of cycles to fracture (NCF) was measured.	OC files significantly outperformed PTG files in all conditions. Both file types showed significant fatigue resistance reduction after 5 min NaOCl immersion. OC files maintained superior performance even after chemical exposure, suggesting better material resilience. PTG-1 min and PTG-5 min showed no significant difference but both were lower than PTG-control.	OC files demonstrated superior cyclic fatigue resistance compared to PTG files. Prolonged NaOCl exposure (5 min) significantly weakened both file types, though OC maintained greater resilience. These findings inform endodontic instrument selection and NaOCl irrigation timing decisions.
Svec [26]	To investigate how simulated clinical conditions (irrigant exposure to saline and NaOCl, sterilization, and cyclic fatigue) affect torsional moment and angular deflection of NiTi rotary endodontic files.	ProFile NiTi rotary files (ISO sizes 25, 30, 35; 25 mm long; 0.04 taper) were tested in saline or 5.25% NaOCl for up to three simulated uses in a 90° curved metal canal (5 mm radius, 1.5 mm diameter) at 150 rpm with ~6 mm axial movement. Five groups were tested: control, saline unsterilized, saline sterilized, NaOCl unsterilized, and NaOCl sterilized.	Torsional moment increased significantly with file size regardless of solution or sterilization. Cyclic fatigue, irrigants, or sterilization showed no consistent impact on torsional moment or angular deflection.	Sterilization and irrigation do not appear to significantly affect file failure behavior.
Kermeoglu [33]	To analyze cyclic fatigue resistance of NiTi rotary files after NaOCl and Irritol (EDTA + CHX final rinse) irrigation, with/without autoclaving.	3 file systems: ProTaper (PTU F2), WaveOne Gold (primary), Reciproc (R25)—70 files per group (25, 0.08 ISO) divided into 7 subgroups (n = 10): control (no treatment), 5.25% NaOCl 5 min at 37 °C, Irritrol 5 min at 37 °C, subgroup 2 + 1-cycle autoclaving, subgroup 4 repeated 3×, subgroup 3 + 1-cycle autoclaving, subgroup 6 repeated 3×. Files tested using X-Smart Plus endomotor until fracture. Data: time to fracture (s), fragment lengths, SEM fracture surface analysis.	Reciproc showed highest cyclic fatigue resistance, ProTaper lowest. Only subgroups 5 and 7 differed significantly from control. Irrigation solutions and single sterilization cycle did not affect fracture time. No significant difference between subgroups 5 and 7. Subgroups 5,6,7 had significantly longer fractured segments versus others.	Immersion in irrigation solutions did not influence cyclic fatigue resistance. However, immersion combined with 3 sterilization cycles significantly decreased fatigue resistance of Reciproc, ProTaper, and WaveOne Gold files.
Pedulla [34]	This study investigates the effects of sodium hypochlorite (NaOCl) immersion and autoclave sterilization on the cyclic fatigue resistance of two types of heat.	210 new NiTi instruments (TF and HyFlex CM) were divided into seven groups (n = 15 each). Groups were treated with combinations of: No treatment (control), Immersion in 5% NaOCl (1 or 3 times), Autoclave sterilization (1 or 3 times), Both treatments (1 or 3 cycles). Instruments were tested in a simulated curved canal for number of cycles to failure (NCF). Surface analysis was done via scanning electron microscopy (SEM) and energy-dispersive X-ray spectroscopy (EDS).	TF instruments showed a significant reduction in fatigue resistance after 3 cycles of sterilization, regardless of NaOCl immersion. HyFlex CM instruments maintained their cyclic fatigue resistance after all treatments. NaOCl immersion alone did not significantly reduce fatigue resistance in either instrument type. HyFlex CM files had a surface oxide layer that may contribute to greater corrosion resistance and durability.	Autoclave sterilization negatively affects TFs after repeated cycles but not HyFlex CM. NaOCl immersion (even repeatedly) has no significant detrimental effect on the cyclic fatigue resistance of the tested NiTi files. HyFlex CM is more resistant to fatigue and better suited for reuse compared to TF under the tested conditions.
Huang [35]	The study examined how high-concentration NaOCl (5.25%) and different temperatures affect cyclic fatigue resistance of 3 NiTi endodontic files: K3, K3XF, and Vortex.	A novel zirconium oxide ceramic canal model simulated clinical conditions. Files were tested in water and 5.25% NaOCl at three temperatures: 22 °C (room), 37 °C (body), and 60 °C (high). Fatigue resistance was measured by rotations until fracture (Nf).	Vortex files showed highest fatigue resistance, followed by K3XF and K3. All files demonstrated decreased resistance at higher temperatures, with lowest at 60 °C. NaOCl (5.25%) caused slightly shorter fatigue life than water (not statistically significant). No corrosion signs observed. Heat-treated files (Vortex, K3XF) performed better due to martensite phase flexibility at clinical temperatures.	High-concentration NaOCl slightly reduced fatigue life but not significantly. Temperature had greater impact on fatigue resistance. Vortex files with higher austenite finish temperature maintained better performance at body temperature (37 °C). Future designs should consider temperature-related NiTi phase transformation properties for improved clinical reliability.
Topcuoglu [36]	To assess how immersion in sodium hypochlorite (NaOCl) affects the cyclic fatigue resistance of 3 different nickel–titanium (NiTi) rotary retreatment instruments: D-RaCe, ProTaper, and Mtwo.	90 new files (30 per brand) were divided into two groups: control (no immersion) and NaOCl immersion (5% NaOCl at 37 °C for 5 min). Cyclic fatigue testing rotated files in artificial canal (60° curvature, 5 mm radius) until fracture. Number of cycles to failure (NCF) was recorded and analyzed.	D-RaCe instruments showed highest cyclic fatigue resistance among the three brands. NaOCl immersion significantly reduced fatigue resistance for D-RaCe but not for ProTaper and Mtwo. No significant difference was observed between ProTaper and Mtwo fatigue resistance. ProTaper showed shorter fracture segments than other brands.	D-RaCe demonstrates superior cyclic fatigue resistance but decreases after NaOCl exposure. Clinicians should consider this when using D-RaCe instruments with NaOCl during retreatment.
Alfawaz [27]	To examine how different NaOCl concentrations and temperatures affect cyclic fatigue resistance of ProTaper Gold (PTG) rotary endodontic instruments made of heat-treated NiTi alloy.	135 PTG instruments were divided into 9 groups exposed to different NaOCl concentrations (distilled water, 2.5%, 5.25%) and temperatures (25 °C, 37 °C, 60 °C). Instruments were rotated in artificial canal until fracture. Number of cycles to fracture (NCF) was measured and fracture surfaces examined using SEM.	Highest fatigue resistance: distilled water at 25 °C. Lowest: 5.25% NaOCl at 60 °C. Increasing NaOCl concentration and temperature both reduced fatigue resistance. Brief NaOCl exposure caused fatigue degradation, especially at higher concentrations.	NiTi instrument performance is significantly affected by irrigant environment, especially NaOCl concentration and temperature. High-temperature, high-concentration NaOCl may increase instrument fracture risk.
Bulem [37]	To investigate whether immersion in sodium hypochlorite (NaOCl) and/or autoclave sterilization affects the cyclic fatigue resistance of four types of nickel–titanium (NiTi) rotary endodontic instruments: ProFile, FlexMaster, Twisted File, Mtwo.	A total of 160 instruments (40 from each brand) were tested. A dynamic cyclic fatigue test was used to simulate clinical use in curved root canals. Instruments were divided into four treatment groups: Control (no treatment), Immersion in 2.5% NaOCl for 5 min, NaOCl + 1 sterilization cycle, NaOCl + 5 sterilization cycles	Mtwo had the highest fatigue resistance (NCF), followed by Twisted File, FlexMaster, and ProFile. Neither NaOCl immersion nor sterilization significantly affected the cyclic fatigue resistance of the instruments (*p* > 0.05). The length of fractured segments also showed no significant difference between groups.	NaOCl immersion and autoclave sterilization (up to 5 cycles) do not adversely affect the cyclic fatigue resistance of the tested NiTi instruments. Among the four brands, Mtwo showed the best resistance to fatigue under all conditions.
Pedulla [38]	To evaluate how NaOCl immersion affects cyclic fatigue resistance of two NiTi endodontic instruments: Reciproc R25 and WaveOne Primary.	Ninety new NiTi instruments (45 Reciproc, 45 WaveOne) were divided into 3 groups: no NaOCl immersion (control), 5% NaOCl immersion for 1 min, and 5% NaOCl immersion for 5 min. All instruments underwent cyclic fatigue testing in simulated curved canal until fracture. Time to fracture (TtF) was recorded.	NaOCl immersion (1–5 min) did not significantly reduce cyclic fatigue resistance. Reciproc R25 showed significantly greater fatigue resistance than WaveOne Primary across all groups. Differences attributed to instrument design and reciprocating mechanics, not alloy composition (both M-wire).	Short-term NaOCl exposure does not degrade cyclic fatigue resistance of M-wire NiTi files. Reciproc R25 shows greater durability than WaveOne Primary, likely due to cross-sectional geometry and mechanical operation.
Cai [39]	To evaluate how common endodontic irrigants (5.25% NaOCl and 17% EDTA) affect surface roughness and cyclic fatigue resistance of two NiTi rotary instruments made from controlled memory (CM) wire: HyFlex and M3.	HyFlex and M3 files were immersed for 10 min in 5.25% NaOCl or 17% EDTA at 37 °C. AFM analyzed surface changes before/after immersion. Fatigue resistance tested via rotational bending in simulated curved canal (45° curve, 3 mm radius).	Surface Roughness: M3 files showed significant roughness increase after both NaOCl and EDTA immersion. HyFlex files showed increased roughness only with EDTA, not NaOCl. Fatigue Resistance: No statistically significant reduction in fatigue resistance for either file type after irrigant immersion. HyFlex files demonstrated higher overall fatigue resistance than M3 files.	Surface damage (increased roughness) occurred in most files after irrigant exposure. Cyclic fatigue resistance was not significantly affected by 10 min irrigant exposure. CM wire instruments retain durability despite short-term exposure to common root canal irrigants.
Cheung [15]	To assess how different environmental conditions (air, water, sodium hypochlorite, and silicone oil) affect the low-cycle fatigue of nickel–titanium rotary instruments.	ProFile NiTi rotary instruments (size 25, 0.04 and 0.06 taper) were tested using custom rotational-bending fatigue machine at 250 rpm until fracture. 4 conditions tested at 23 °C: air, deionized water, 1.2% NaOCl, and silicone oil. Curved instrument sections were immersed in medium while constrained by adjustable steel pins. Fracture cycles, curvature radius, and fracture diameter were measured.	Instruments in corrosive environments (water, hypochlorite) showed steeper slopes versus non-corrosive environments (air, silicone oil). Hypochlorite produced shortest fatigue life, while air and silicone oil showed similar results. Crack initiation sites differed significantly between groups (*p* < 0.05), with hypochlorite favoring single crack origins.	Environmental conditions significantly affect NiTi instrument fatigue behavior, with sodium hypochlorite being most detrimental to fatigue life.
Elnaghy [40]	To evaluate how different environmental conditions (NaOCl irrigant, saline versus air) affect fatigue life of two modern reciprocating endodontic file systems at physiological temperature.	180 single-use files (90 WaveOne Gold 0.25/0.07 and 90 Reciproc 0.25/0.08) were divided equally across 3 testing conditions: dry air at room temperature, physiological saline at body temperature, and 5% bleach solution at body temperature.	Gold-treated files outperformed standard alloy files in all environments. Both file types lasted longer when tested dry versus liquid immersion. Corrosive bleach and neutral saline caused similar life reductions. Gold files survived 1027 cycles with 99% confidence when dry, while standard files in bleach showed poorest performance at 613 predicted cycles.	Liquid environments reduce reciprocating file working life compared to dry conditions. Gold heat-treated instruments demonstrated superior durability over standard alloy files due to enhanced metallurgy and design improvements.
Keles [41]	To assess how elevated temperatures of sodium hypochlorite irrigant affect the fatigue durability of modern heat-treated reciprocating endodontic file systems.	5 file systems (n = 10 each) were tested: Reciproc, Reciproc Blue, WaveOne, WaveOne Gold, and OneShape. Each underwent fatigue testing in five conditions: dry control, distilled water (37 °C and 60 °C), and 5.25% sodium hypochlorite (37 °C and 60 °C). Files were immersed for 5 min before testing in a standardized 60-degree curved artificial canal (5 mm radius) using pecking motion simulation until fracture. Time to failure and fragment lengths were recorded.	Reciproc Blue showed superior fatigue resistance across all conditions. Sodium hypochlorite at 60 °C significantly reduced fatigue life for all systems except Reciproc Blue, while hot distilled water (60 °C) improved Reciproc Blue’s performance. Heat-treated instruments outperformed conventional superelastic files. WaveOne showed the greatest reduction after hot sodium hypochlorite exposure; OneShape also degraded at higher temperatures.	Preheated sodium hypochlorite significantly reduces fatigue resistance of reciprocating files. Heat-treated instruments showed superior durability versus conventional files.
Alfawaz [42]	To investigate how irrigating solutions of EDTA and sodium hypochlorite affect the fatigue durability of EdgeTaper Platinum files compared to ProTaper Gold instruments.	120 instruments (60 ProTaper Gold F2 and 60 EdgeTaper Platinum F2, both 25/0.08) were divided into three groups per system (n = 20): 17% EDTA, 5.25% NaOCl, and distilled water control. Fatigue testing used 60-degree curved canals (5 mm radius) with instruments immersed in test solutions at 37 °C. Instruments rotated at 300 rpm until fracture; cycles to failure were calculated and fracture surfaces examined.	EdgeTaper Platinum demonstrated higher cycles to failure than ProTaper Gold across all solutions. Sodium hypochlorite dramatically reduced fatigue resistance compared to water and EDTA. EDTA showed no impact versus distilled water. EdgeTaper Platinum achieved 50% better fatigue resistance than ProTaper Gold in all conditions.	EdgeTaper Platinum showed superior fatigue performance versus ProTaper Gold in all irrigation solutions. EDTA did not affect instrument durability, while sodium hypochlorite significantly reduced lifespan of both systems.
Tyagi [43]	To assess how different concentrations and temperatures of sodium hypochlorite irrigant affect the fatigue durability of 4 different heat-treated nickel–titanium rotary file systems.	720 instruments were divided into 4 groups (n = 180): GenEndo, ProTaper Gold, Hero Gold, and EdgeFile X3. Each group had 9 subgroups (n = 20) based on three solutions (distilled water, 2.5% NaOCl, 5.25% NaOCl) at three temperatures (25 °C, 37 °C, 60 °C). Fatigue testing used 60-degree curved canals (5 mm radius) with instruments rotating at 300 rpm until fracture.	GenEndo showed superior fatigue resistance, followed by ProTaper Gold, Hero Gold, and EdgeFile X3. Sodium hypochlorite significantly reduced fatigue life versus water, with higher concentrations causing greater degradation. Increasing temperature (25 °C to 37 °C to 60 °C) progressively decreased resistance. GenEndo remained stable across conditions while other systems showed significant changes.	Sodium hypochlorite concentration and temperature significantly impact the fatigue resistance of modern NiTi rotary instruments. GenEndo files showed overall highest fatigue resistance.
Erik [44]	Comparison of the effect of etidronic acid (HEBP), NaOCl, and EDTA on cyclic fatigue resistance of NiTi instruments.	300 NiTi instruments (RPC Blue, HyFlex EDM, WaveOne Gold) were tested in artificial canals milled in CEREC blocks. Five groups were tested: distilled water (control), 6% NaOCl, 17% EDTA, 18% HEBP, and 3% NaOCl + 9% HEBP. Files rotated until fracture with time recorded in seconds and cycles to fracture (NCF) calculated.	HyFlex EDM showed significantly higher cyclic fatigue resistance than WaveOne Gold and RPC Blue in all conditions. No difference was found between WaveOne Gold and RPC Blue. All files showed significantly lower fatigue resistance in group 5 (3% NaOCl + 9% HEBP) versus other groups.	EDTA, NaOCl, and HEBP solutions alone did not affect cyclic fatigue resistance. However, combined NaOCl and HEBP significantly reduced fatigue resistance of all tested files. HyFlex EDM consistently showed superior cyclic fatigue resistance versus RPC Blue and WaveOne Gold across all conditions.
Pedulla [45]	Examination of the resistance to cyclic fatigue of NiTi files after immersion in NaOCl.	150 NiTi instruments (Twisted Files, Revo S SU, Mtwo, 25.06) were tested: 50 files per brand randomly assigned to 5 groups (n = 10). Group 1: control; Groups 2–5: 5% NaOCl at 37 °C for 16 mm. Group 2: static 5 min; Group 3: static 1 min; Group 4: dynamic 300 rpm 5 min; Group 5: dynamic 300 rpm 1 min. Time to fracture and cycles to failure were recorded.	NaOCl immersion did not significantly affect cyclic fatigue resistance. Twisted Files showed higher resistance than Revo S SU in all groups. No significant differences were found between Twisted Files vs. Mtwo or Mtwo vs. Revo S SU. Statistically significant differences existed between brands within the same groups.	Static or dynamic NaOCl immersion (1–5 min) did not significantly reduce cyclic fatigue resistance. However, instrument type influenced resistance: Twisted Files showed superior cyclic fatigue resistance versus Mtwo and Revo S SU.
Dosanjh [46]	Examination of the effect of temperature changes on the cyclic fatigue of Ni-Ti rotary files.	120 NiTi instruments (#25, 0.04 taper, 25 mm) were divided into 3 groups: EF (EdgeFile), VB (Vortex Blue), ESX. Files were tested in simulated canals at 4 temperatures (30 files/cycle): 3 °C, 22 °C, 37 °C, 60 °C. Cycles to fracture were calculated.	VB showed significant decrease in cycles to fracture from 3 °C to 60 °C. ESX showed significant decrease from 3 °C to 37 °C. EF showed significant increase from 3 °C to 22 °C, then significant decrease from 22 °C to 37 °C. At each temperature, EF > VB > ESX for cycles to fracture.	Temperature significantly affected cyclic fatigue of NiTi instruments. At each temperature, cycles to fracture ranked: EF > VB > ESX.

**Table 3:** JBI checklist for quasi-experimental studies (nonrandomized experimental studies).

Authors	1. Is It Clear in the Study What Is the ‘Cause’ and What Is the ‘Effect’?	2. Were the Participants Included in Any Comparisons Similar?	3. Were the Participants Included in Any Comparisons Receiving Similar Treatment/Care, Other than the Exposure or Intervention of Interest?	4. Was There a Control Group?	5. Were There Multiple Measurements of the Outcome Both Pre and Post the Intervention/Exposure?	6. Was Follow up Complete and if Not, Were Differences Between Groups in Terms of Their Follow Up Adequately Described and Analyzed?	7. Were the Outcomes of Participants Included in Any Comparisons Measured in the Same Way?	8. Were Outcomes Measured in a Reliable Way?	9. Was Appropriate Statistical Analysis Used?
de Castro Martins [22]	Yes	Yes	Yes	Yes	Yes	Yes	Yes	Yes	Yes
Cheung [28]	Yes	Yes	Yes	No	No	Yes	Yes	Yes	Yes
Ormiga Galvão Barbosa [23]	Yes	Yes	Yes	Yes	No	Yes	Yes	Yes	Yes
Berutti [24]	Yes	Yes	Yes	Yes	No	Yes	Yes	Yes	Yes
Javadi [29]	Yes	Yes	Yes	Yes	No	Yes	Yes	Yes	Yes
Palma [30]	Yes	Yes	Yes	Yes	No	Yes	Yes	Yes	Yes
Priya [31]	Yes	Yes	Yes	Yes	No	Yes	Yes	Yes	Yes
Mousavi [25]	Yes	Yes	Yes	Yes	Yes	Yes	Yes	Yes	Yes
Tanomaru [47]	Yes	Yes	Yes	Yes	No	Yes	Yes	Yes	Yes
Abuhulaibah [32]	Yes	Yes	Yes	Yes	No	Yes	Yes	Yes	Yes
Svec [26]	Yes	Yes	No	Yes	No	Yes	Yes	Yes	Yes
Kermeoglu [33]	Yes	Yes	Yes	Yes	No	Yes	Yes	Yes	Yes
Pedulla [34]	Yes	Yes	Yes	Yes	No	Yes	Yes	Yes	Yes
Huang [35]	Yes	Yes	Yes	Yes	No	Yes	Yes	Yes	Yes
Topcuoglu [36]	Yes	Yes	Yes	Yes	No	Yes	Yes	Yes	Yes
Alfawaz [27]	Yes	Yes	Yes	Yes	No	Yes	Yes	Yes	Yes
Bulem [37]	Yes	Yes	Yes	Yes	No	Yes	Yes	Yes	Yes
Pedulla [38]	Yes	Yes	Yes	Yes	No	Yes	Yes	Yes	Yes
Cai [39]	Yes	Yes	Yes	Yes	No	Yes	Yes	Yes	Yes
Cheung [15]	Yes	Yes	Yes	Yes	No	Yes	Yes	Yes	Yes
Elnaghy [40]	Yes	Yes	Yes	Yes	No	Yes	Yes	Yes	Yes
Keles [41]	Yes	Yes	Yes	Yes	No	Yes	Yes	Yes	Yes
Alfawaz [42]	Yes	Yes	Yes	Yes	No	Yes	Yes	Yes	Yes
Tyagi [43]	Yes	Yes	Yes	Yes	No	Yes	Yes	Yes	Yes
Erik [44]	Yes	Yes	Yes	Yes	No	Yes	Yes	Yes	Yes
Pedulla [45]	Yes	Yes	Yes	Yes	No	Yes	Yes	Yes	Yes
Dosanjh [46]	Yes	Yes	Yes	Yes	No	Yes	Yes	Yes	Yes

## Data Availability

No new data were created or analyzed in this study. Data sharing is not applicable to this article..
